# Lineage-specific expansions of polinton-like viruses in photosynthetic cryptophytes

**DOI:** 10.1186/s40168-025-02148-0

**Published:** 2025-07-01

**Authors:** Paul-Adrian Bulzu, Helena Henriques Vieira, Rohit Ghai

**Affiliations:** Department of Aquatic Microbial Ecology, Institute of Hydrobiology, Biology Centre of the Czech Academy of Sciences, České Budějovice, Czech Republic

**Keywords:** Polinton-like viruses, Virophages, Cryptophytes, Chrysophytes, Rhodomonas

## Abstract

**Background:**

Polinton-like viruses (PLVs) are diverse eukaryotic DNA viral elements (14–40 kb) that often undergo significant expansion within protist genomes through repeated insertion events. Emerging evidence indicates they function as antiviral defense systems in protists, reducing the progeny yield of their infecting giant viruses (phylum *Nucleocytoviricota*) and influencing the population dynamics and evolution of both viruses and their hosts. While many PLVs have been identified within the genomes of sequenced protists, most were recovered from metagenomic data. Even with the large number of PLVs identified from metagenomic data, their host-virus linkages remain unknown owing to the scarcity of ecologically relevant protist genomes. Additionally, the extent of PLV diversification within abundant freshwater taxa remains undetermined. In order to tackle these questions, high-quality genomes of abundant and representative taxa that bridge genomic and metagenomic PLVs are necessary. In this regard, cryptophytes, which are among the most widely distributed, abundant organisms in freshwaters and have remained largely out of bounds of genomic and metagenomic approaches, are ideal candidates for investigating the diversification of such viral elements both in cellular and environmental context.

**Results:**

We leveraged long-read sequencing to recover large (200–600 Mb), high-quality, and highly repetitive (> 60%) genomes of representative freshwater and marine photosynthetic cryptophytes. We uncovered over a thousand complete PLVs within these genomes, revealing vast lineage-specific expansions, particularly in the common freshwater cryptophyte *Rhodomonas lacustris.* By combining deep sequence homology annotation with biological network analyses, we discern well-defined PLV groups defined by characteristic gene-sharing patterns and the use of distinct strategies for replication and integration within host genomes. Finally, the PLVs recovered from these cryptophyte genomes also allow us to assign host-virus linkages in environmental sequencing data.

**Conclusions:**

Our findings provide a primer for understanding the evolutionary history, gene content, modes of replication and infection strategies of cryptophyte PLVs, with special emphasis on their expansion as endogenous viral elements (EVEs) in freshwater bloom-forming *R. lacustris*.

Video Abstract

**Supplementary Information:**

The online version contains supplementary material available at 10.1186/s40168-025-02148-0.

## Background

Eukaryotic genomes often contain endogenous viral elements (EVEs) and mobile genetic elements (MGEs) which can together account for a substantial proportion of total genome length [163]. Such genomic parasites can move (“jump”) in or out of the host genome, with the distinctive feature that MGEs do not encode for structural proteins such as capsids which would allow them to propagate independently of their host [73, 128].

Polinton-like viruses (PLVs) are a prominent group of EVEs commonly identified in eukaryotic genomes, with the notable exception of mammals and land plants [6]. These 14–40 kb double-stranded DNA elements encode capsid proteins and are evolutionarily related to the Maverick–Polinton class of self-synthesizing transposons, defined by the presence of a protein-primed family B DNA polymerase (PolB), a retroviral-type integrase (rve-INT), and terminal inverted repeats (TIRs) [13, 67, 119]. PLVs often retain these hallmark features, including TIRs several hundred base pairs long, and are sometimes flanked by short 5–6 bp target site duplications (TSDs) which arise upon integration into the host genome [14, 42, 159].

Phylogenetic and structural analyses indicate that PLVs evolved from bacterial tectiviruses [77] and they are related to both virophages and eukaryote-infecting giant viruses of the nucleocytoplasmic large DNA virus (NCLDV) group, which virophages are known to parasitize [6, 7, 129]. Despite their divergence, PLVs and NCLDV both retain a conserved morphogenetic gene module, including single- and double-jelly-roll capsids, a capsid maturation protease, and a DNA packaging ATPase [7, 78]. These shared genes support their common origin and inclusion within the viral kingdom *Bamfordvirae*, [7, 74, 129]. Recent taxonomic proposals place most, if not all, PLVs within the class *Polintoviricetes* [129]. In this study, we use “PLVs” to refer to both accepted and prospective members of this class, including MCP-encoding polintons, while reserving the term “virophage” for members of *Maveriviricetes*, which encode distinct capsid proteins and depend upon co-infection with giant viruses for replication [129].

Recent experimental evidence suggests that PLVs, like virophages, may suppress giant virus proliferation thus acting as antiviral agents in protists [76, 128]. While the virophage lifestyle is reasonably well-characterized, thanks to several established protist—giant virus—virophage co-culture systems [44, 47, 56, 81, 136], only two PLVs have been experimentally characterized in culture to date: PgVV Gezel-14 T (19 kb) which parasitizes the nucleocytovirus PgV-14 T of haptophyte *Phaeocystis globosa * [128]*,* and *Tetraselmis* virus N1 (TsV-N1; 31 kb) capable of inducing autonomous lytic infections in the marine chlorophyte *Tetraselmis striata* [111]. Additional PLVs have been isolated from protists, and some have even been reported active in animals. For instance, three PLVs—Curly, Larry, and Moe—were co-isolated with the giant mimivirus CpV-BQ2 during infection of freshwater haptophyte *Chrysochromulina parva* [149]. PLV-like elements have also been found in association with entomopoxviruses infecting lepidopteran insects, establishing the first known link between PLVs and poxviruses [9]. Recent studies identified endogenous PLVs in the genomes of stony corals (*Cnidaria*) [147] and in *T. striata*, providing direct evidence that TsV-N1 persists as both a free virus and integrated EVE [27].

To date, most PLV genomes have been assembled from short-read metaviromes [14], which provide very limited information regarding host identity and viral lifestyle. However, recent screening of publicly available short-read-sequenced protist genomes revealed a vast collection of PLVs and virophages integrated into their nuclear genomes [13]. This enabled host assignment for many PLVs previously recovered as free viruses. The same study highlighted the advantages of long-read sequencing in resolving long insertions of EVEs present in many similar copies, revealing a wide variability in their abundance across different protists. Thus, while some small eukaryotes (e.g., *Ostreococcus* spp.) contain only a few insertions, others—such as dinophytes (e.g., *Polarella glacialis*) and rhizarians (e.g., *Paulinella micropora*)—harbor thousands, accounting for over 10% of their nuclear genome. In the extreme case of *Trichomonas vaginalis*, Mavericks-Polintons comprise over 30% of their total genomic length [13]

Among cryptophyte algae, *Guillardia theta* was the first species to have its genome fully sequenced [31] and one of the first protists in which PLVs were identified as EVEs [166, 167]. However, only three complete and two partial PLVs have been detected in its genome. Similarly, five partial PLVs were reported in the plastid-lacking cryptophyte *Goniomonas avonlea* [26], and until recently, these were the only cryptophyte-associated PLVs known. Aside from their relatively small genome sizes (*G. theta—*87.1 Mb, *G. avonlea*—91.5 Mb), the underrepresentation of EVEs within these protists may be explained by technical limitations associated with short-read sequencing [13, 55].

Cryptomonads are small (5–50 μm), biflagellate unicellular algae ubiquitously distributed in both marine and freshwater habitats [52, 59, 142]. They include plastid-bearing (*Cryptophyceae*), as well as plastid-lacking species (*Goniomonadaceae*). Photosynthetic cryptophytes acquired their plastids through secondary endosymbiosis with a red alga and retain a vestigial nucleus from the endosymbiont, known as the nucleomorph [31, 32, 124]. The aplastidic state in *Goniomonadaceae* is considered ancestral, rather than being the result of plastid loss [26]. In freshwater environments, photosynthetic cryptophytes contribute significantly to spring blooms, whereas aplastidic forms (e.g., CRY1 lineage) are key bacterivores throughout the water column [52, 68, 142]. Aside from abiotic factors and grazing by zooplankton, the involvement of viruses in bloom dynamics was first suggested by the detection of metagenomic fragments from giant viruses during bloom collapse [68], followed by the successful isolation of *Budvirus*, a giant virus infecting *Rhodomonas lacustris * [155].

Here, we apply long-read sequencing and assembly to generate high-quality reference genomes for five protists sequenced in this study—four cryptophytes and one ochrophyte. These groups were selected as part of an ongoing exploratory effort to investigate the genomics and transcriptomics of major protist lineages that play key roles in aquatic ecosystems. Cryptophytes and chrysophytes (*Ochrophyta* clade) are widespread and often reach high abundances during seasonal blooms, yet genomic resources for them remain scarce. In addition to our newly sequenced isolates, we reassembled four publicly available ochrophyte genomes to improve their contiguity and to reduce contamination, as several of the original assemblies were highly fragmented and contained non-eukaryotic sequences. We also assembled two additional cryptophyte genomes de novo from public long-read datasets that lacked published assemblies. This resulted in a total of 11 high-confidence protist genomes spanning both freshwater and marine species. Although previous studies have linked PLVs to protist hosts using short-read data, and to a lesser extent long-read data [13], many PLVs in cryptophytes appeared fragmented or degraded. This has made it difficult to resolve their gene content and genomic context. Our study complements these efforts by using long-read sequencing to more accurately reconstruct PLVs and correctly place them within host genomes, allowing for a more comprehensive assessment of their structure, gene repertoire, and evolution.

To interpret our findings in a broader context, we also performed large-scale comparative analyses across the genomic sequences of more than 8,000 PLV and related elements. This dataset includes newly recovered elements from the 11 genomes analyzed here, previously published ones [13] [14], and sequences from PLVs identified as free viral entities [111, 128, 149]. This integrative approach enabled us to explore PLV diversity and genome architecture across a wide range of eukaryotic lineages. Our data reveal vast lineage-specific expansions of PLVs within cryptophytes and offer new insights into their genomic content and evolutionary relationships.

## Results and discussion

### Genome reconstruction

To explore the mobile genetic element landscape of cryptophyte algae, a total of six ubiquitous photosynthetic strains from this class were assembled from long-read datasets produced in this study (*n* = 4)—freshwater *Rhodomonas lacustris* (NIVA-8/82) and *Cryptomonas pyrenoidifera* (NIVA-2/81), and the marine strains *Storeatula* sp. (K-1488) and *Rhinomonas nottbecki* (K-1855)—and from sequencing data made available in public repositories (*n* = 2)—freshwater *Cryptomonas borealis* (NIES-276) and marine *Rhodomonas baltica* (CCAP 979/9) (Supplementary Table S1).

Additionally, the genomes of five widespread freshwater chrysophytes were reconstructed here using long-reads. Four of them were reassembled from publicly available data with the purpose of improving overall genome contiguity by using updated assembly methods and to reduce contamination by non-eukaryotic sequences in non-axenically grown protists: *Pedosmpumella encystans* (JBM-S11), *Poterioochromonas* (DS), *Poteriospumella lacustris* (JBM10) and *Hydrurus foetidus* [96] (see Supplementary Table S1). Additionally, a fifth, high-quality genome, was assembled for *Ochromonas danica* (SAG 933–7) from data generated in this study by applying two complementary long-read sequencing approaches (i.e., PacBio HiFi and Oxford Nanopore).

Initial comparisons between genomes with high estimated completeness (i.e., BUSCO > 75%) revealed a nearly ten-fold size difference between ochrophytes, usually falling below 50 Mbp, and cryptophyte genomes that sometimes surpassed 500 Mbp (Supplementary Table S2). Such discrepancy in haploid genome sizes could be partially explained by genomic repeat content which often accounts for more than 50% of recovered cryptophyte genomes versus only 20% in most analyzed chrysophytes (Supplementary Fig. S1). Multiple telomere-to-telomere chromosomes as well as centromere-to-telomere fragments were recovered from sequenced genomes, particularly those of *R. lacustris* and *O. danica,* confirming the successful resolution of repetitive genomic stretches.

### Recovery of polinton-like viruses and virophages

A search for integrated (i.e., endogenous) PLVs/virophages was carried out across all reconstructed genomes (*n* = 11) by scanning for multiple hallmark features such as genes encoding for double-jelly roll major capsids (DJR-MCPs) typical of PLVs and virophages, consistent GC composition shifts across long genomic stretches (~ 5–80 kb), the presence of genes known to be enriched within PLVs/virophages (i.e., DNA packaging ATPase, integrases, capsid maturation protease), and positive matches to known Maverick-Polinton elements from the DFAM database (see the “Material and methods” section). Complete PLVs/virophages were recognized by the presence of flanking TIRs consisting of hundred-to-thousands base pair long stretches of DNA, sometimes bordered by short (~ 4–6 bp) direct repeats resulting from integrase activity and known as target site duplication (TSDs). Overall, we retrieved 1193 complete endogenous small viral-like genomes within cryptophytes and chrysophytes (1175 PLVs, 16 virophages and two hybrid elements containing both virophage-like and PLV MCPs; Supplementary Table S3). Among cryptophytes, vast expansions of PLVs (mean length 22.2 kb) were identified in *R. lacustris* (*n* = 706)*,* comprising 4.75% (2.1 PLVs per Mb) of the total recovered genome length (331 Mb). A proportionately high number of PLVs (*n* = 57) were identified in the partial genome (~ 5%) of *C. pyrenoidifera* accounting for over 1.7% of the total recovered length while the large genomes (> 500 Mb) of closely related *R. nottbecki* and *Storeatula* sp. harbored significantly fewer integrated PLVs (*n* = 57 and *n* = 101, respectively). Surprisingly, virophage-type MCP genes were not detectable within analyzed cryptophytes indicating either their real absence or the use of distant homologues that could not be discerned by available models. Detailed genomic information, including TIR and TSD conservation is included in Supplementary Table S4 and Supplementary Fig. S2. Large deviations in GC content between endogenized PLVs and host genome are typical within all studied cryptophytes (e.g., highest difference in *C. borealis*—28% to lowest in *R. lacustris*—14%) (Supplementary Fig. S2c). The level of sequence conservation (% identity) between pairs of TIRs flanking each element is indicative of recent mobilization [29]. The highest number of conserved TSDs was found among *R. lacustris* PLVs (34%), followed by *C. borealis* (22%), although TIR conservation is highest in *Storeatula* (Supplementary Table S4, Supplementary Fig. S2f).

### Phylogenetic analysis of major capsid proteins

**Fig. 1:**
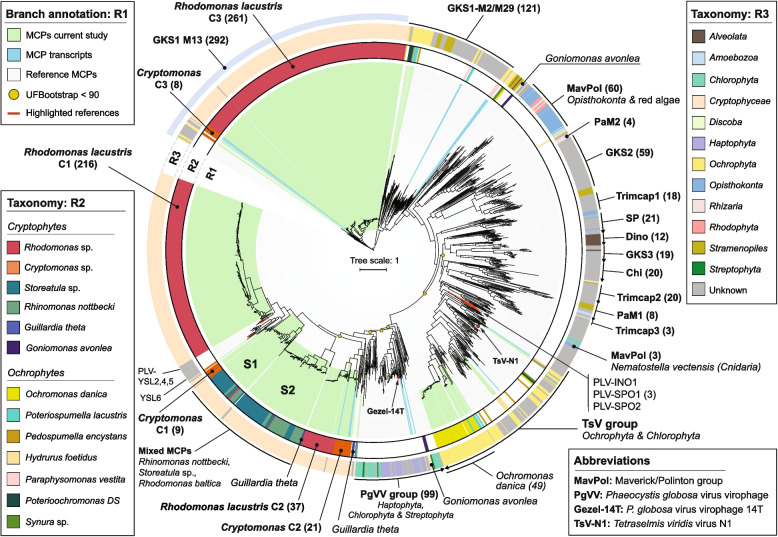
Maximum-likelihood phylogenetic tree of PLV major capsid proteins (MCPs). The unrooted tree includes complete MCPs recovered from PLV genomes recovered in this study and a comprehensive collection of reference sequences collected from previously published genomic, metagenomic and transcriptomic data. PLVs recovered in this study are highlighted with green in ring R1 and RNA transcripts with blue. Taxonomy is indicated in the outer rings: R2—genus-level taxonomy and R3—major clades of eukaryotes. Ultrafast bootstrap (UFB) values are indicated for major branches where the values fall below 90. The other major clades are strongly supported (≥ 90)

A phylogenetic tree of major capsid protein (MCP) sequences recovered from complete PLVs identified in cryptophyte (*n* = 677) and ochrophyte (*n* = 63) genomes, along with a representative collection of sequences from published datasets (*n* = 743; see the “Material and methods” section) revealed that cryptophyte-associated PLVs form two large and well-supported clades (branches highlighted in green, Fig. 1). These clades are positioned at opposite ends of the unrooted tree (Fig. 1) and contain PLVs infecting the same host genera (i.e., *Rhodomonas* and *Cryptomonas*), suggesting independent acquisition events.

The basal clade brings together PLVs of the Gossevirus type (GKS1, Fig. 1 and Supplementary Fig. S3a), which were initially reported in an alpine lake and shown to be a distinct group of viruses based on MCP clustering and gene-sharing networks [14]. All cryptophyte capsids included in this clade were consistently detected by the same HMM model (M13) which also covered the most basal chrysophyte reference sequences (*Chromulina, Chromulinospumella, Dinobryon*) (Fig. 1, GKS1-M13). The previously defined GKS1 group is split here into two distinct subclades (Fig. 1, GKS1-M2/M29) bringing together MCPs from ochrophytes and oomycetes, largely covered by four HMM models: M2, M29 for the first, and M32, M71 for the latter. Since only a subset of representative, high-quality (i.e., non-fragmented and non-redundant) sequences was included in the phylogenetic analysis, scans using HMM M13 across the whole collection of PLVs revealed that 45% (*n* = 317) of *R. lacustris* PLVs belong to the GKS1 clade, while they remain very scarce across other cryptophytes. Other occurrences were noted in *C. pyrenoidifera*, representing 10.5% (*n* = 6) of all recovered PLVs; *C. borealis*, with 3.3% (*n* = 6); and only one GKS1 PLV of ~ 28 kb in *O. danica*.

The other cryptophyte clade located at the tip of the tree (Fig. 1), which appears also to be more diverse (Supplementary Fig. S3b), contains two well-supported subclades encompassing freshwater *R. lacustris* and *Cryptomonas* MCP groups, with interspersed sequences from marine cryptophytes. Previously described PLVs YSL2, YSL4 and YSL5, recovered from Yellowstone lakes metagenomes during one of the first PLV studies [166, 167], formed a cluster with complete ultrafast bootstrap support (UFB = 100) within the terminal *R. lacustris* clade (*n* = 216). In turn, these two groups neighbor a sister clade containing *Cryptomonas*-affiliated sequences and the MCP of PLV YSL6. Two sister clades (further S1 and S2), each containing a collection of *R. nottbecki*, *Storeatula,* and *R. baltica* MCPs neighour and respectively precede the terminal *Rhodomonas-Cryptomonas* cluster. All three hosts are closely related marine plastidic cryptophytes that can co-occur in the environment [95]. Two reference PLV MCPs from marine *G. theta*, also a photosynthetic cryptophyte, form a sister clade with the more basal S2 group. Only one subgroup of *R. nottbecki* MCPs is pure with almost identical sequences while all others are mixed with *Storeatula*-associated PLVs. This in turn suggests that both strains may be infected by the same PLVs, showing a broad host range. Conversely, throughout the phylogenetic tree, mixing of MCPs does not occur between *Cryptomonas* and *Rhodomonas* subclades, which is consistent with the increased phylogenetic divergence of their hosts.

The most basal subclade of the large terminal cryptophyte cluster (Supplementary Fig. S3a) brings together freshwater *Cryptomonas* (*n* = 21) and *Rhodomonas* (*n* = 37) MCPs. Marine *G. theta* (*n* = 2) and metagenomic PLV SAF2 and SAF3 capsid sequences form a sister clade with this subgroup. One MCP of *R. nottbecki* and one of *Storeatula* sp. group together outside of the major terminal cluster, albeit with low support (UFB = 63). The first non-cryptophyte sister clade brings together PLV MCPs from green algae and plants (*n* = 21) as well as marine metagenomic PLVs ACE1 and SAF5. Although this association has relatively low support (UFB = 82), it does hint towards Viridiplantae as the potential origin of cryptophyte PLV MCPs. The MCPs of aplastidic cryptomonad *G. avonlea* (*n* = 5) clustered with animal polintons (*n* = 2) and with freshwater green algae (*n* = 3) within the PgVV group.

### Distinct types of PLVs may co-occur in the same host genome

**Fig. 2:**
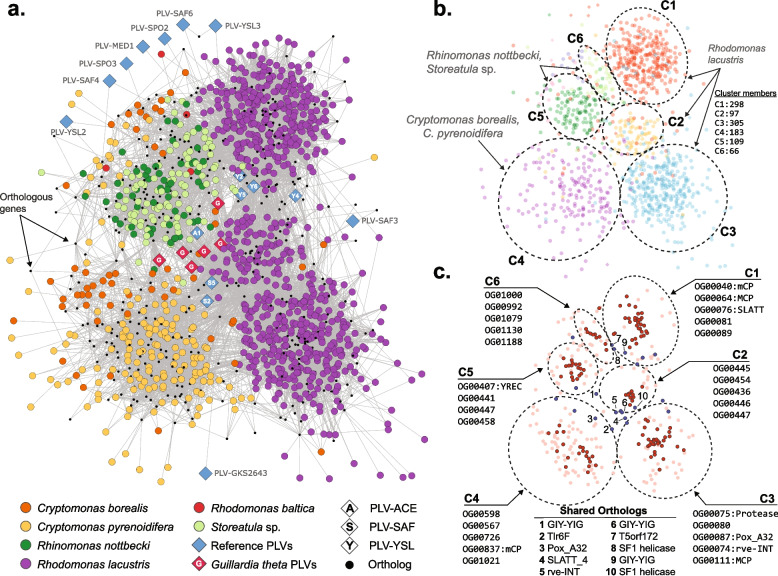
Bipartite network of gene sharing among cryptophyte PLVs. **a** PLVs recovered from cryptophyte genomes in this study are shown as circles, colored according to host taxonomy. Reference genomes are depicted as blue diamonds, with *G. theta* highlighted in red. Shared genes are represented as small black dots. **b** PLV clusters are highlighted using dashed circles. **c** Independent representation of shared orthologous genes underlying the clustering patterns of PLV genomes. Orthologs connected to ≥ 30 PLVs are highlighted in dark red; those with fewer connections appear in light red. Orthologs linked to fewer than 10 PLVs are not shown. The five most connected orthologs are listed at the margins. Highly connected gene clusters (> 30 connections) are indicated with blue dots

To determine gene-sharing patterns between PLVs affiliated with cryptophytes and how these patterns may define clusters with particular attributes we constructed a bi-partite network [30] by connecting individual genomes to orthologous genes they encode (Fig. 2a). By employing community detection methods we were able to define six distinct clusters (Fig. 2b, Supplementary Table S5), as a complementary approach to the single-marker (i.e., MCP) phylogenetic analysis. One significant advantage of this method is its ability to define PLV genome clusters using unannotated proteins, which is often the case with viral genomes. Established bi-partite clusters largely matched the ones observed in the phylogenetic tree and showed significant overlap with them. In this sense, the three large phylogenetic *R. lacustris* MCP groups (Fig. 1) are represented in the network by clusters C1-C3 (Fig. 2b), with C1 corresponding to the terminal clade of the phylogenetic tree (Fig. 1, Supplementary Fig. 2b), C2 to the smaller *R. lacustris* group at the base of the large terminal cryptophyte cluster (Supplementary Fig. S3a), and C3 with the basal group of GKS1 viruses.

Only a few orthologous protein-encoding genes (Fig. 2c) could be annotated by deep homology searches (see the “Material and methods” section). Most orthologs that could be associated with known protein domains were the ones shared among clusters, thus highlighting the conserved “core” genes in the different subtypes of PLVs. It is also expected that different families of the same gene, such as the subtypes of MCP, drive individual cluster formation.

### Genomic content of the polinton-like viruses

**Fig. 3:**
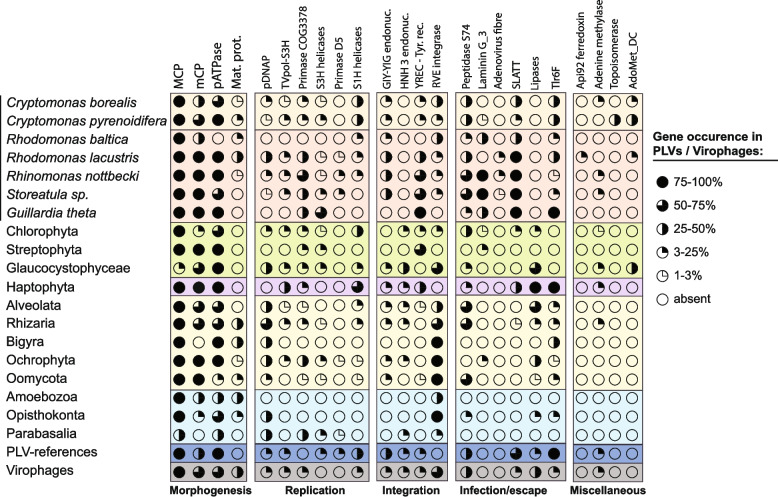
Gene content of PLVs and virophages across major taxonomic groups. Circles are filled according to the percentage of PLVs from each eukaryotic group or specific organism that encodes the indicated gene. Categories of genes are broadly defined according to their expected function. Color shading is used to indicate closely related eukaryotes. Only complete PLVs (with TIRs) were included

**Fig. 4:**
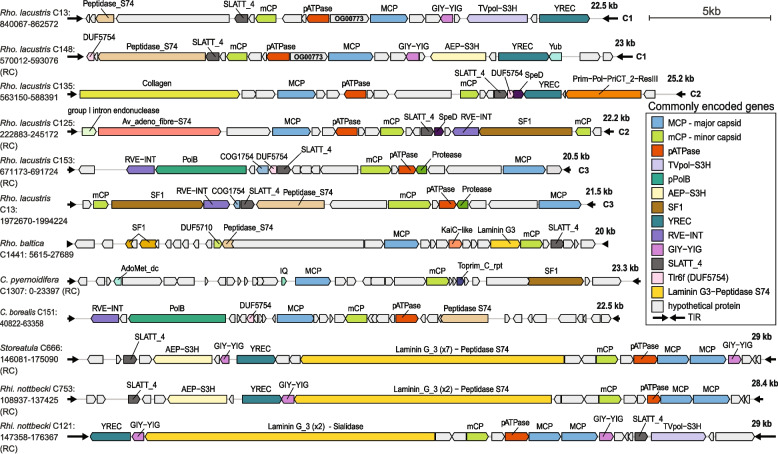
Representative polinton-like viruses recovered from cryptophyte hosts. Critical genes involved in viral morphogenesis and genome replication, integration into the host’s genome, and infection are described in the main text. Hypothetical proteins are shaded in light gray. All depicted PLVs are complete, having conserved terminal inverted repeats (TIRs). Host genomic locations for each PLV are indicated by contig number and genomic region. Abbreviations: *Rho. lacustris—Rhodomonas lacustris*, *Rho. baltica—Rhodomonas baltica*, *C. pyrenoidifera—Cryptomonas pyrenoidifera*, *C. borealis—Cryptomonas borealis*, *Rhi. nottbecki—Rhinomonas nottbecki*. Cluster membership for *R. lacustris* contigs is indicated at the end of each genomes (C1-C3)

To begin to unravel aspects of PLV biology based on their gene content we annotated all predicted proteins from polinton-like elements included in our collection. Three out of four genes comprising the critical morphogenetic module, namely major and minor capsid proteins (MCP and mCP, respectively), and DNA packaging ATPase (pATPase) were conserved within most recovered cryptophyte and ochrophyte PLVs (Fig. 3; Fig. 4; Supplementary Table S6). A morphogenetic gene encoding for cysteine protease, involved in capsid maturation, was detected only in ochrophyte virophages (*n* = 6) and in cluster C3 of *R. lacustris*, where it was present in almost all members (277 of 305 PLVs). This is consistent with findings reported for the GKS1 (Gossevirus) group of PLVs from alpine lakes, where cysteine protease was identified as a core gene [14]. According to the recently updated classification, a reduced morphogenetic gene module is typical of the *Aquintoviricetes* clade of polinton-like viruses, while the complete one is common in Polintoviricetes [74]. Surprisingly, most *Storeatula* (*n* = 54) and *R. nottbecki* (*n* = 50) PLVs were found to harbor two adjacent MCP genes which encode full-length capsid proteins, as noted before in the case of *G. theta* PLV-3 [166, 167].

### Alternative genes mediate integration within host genomes

Two distinct classes of unrelated integrases were detected within the cryptophyte PLVs analyzed here: the retroviral-type integrase (rve-INT), characteristic of polintons [16] and Mavirus-like virophages [44], and tyrosine recombinase (YREC), typically found in virophages related to Sputnik and Zamilon [81], as well as in a group of eukaryotic DNA transposons from fungi known as *Cryptons* [49].

While rve-INT typically generates 6-bp-long target site duplications (TSDs) upon insertion [43], tyrosine recombinases lack this activity [116]. Instead, they mediate site-specific recombination of circular donor DNA [35], suggesting that PLVs encoding them also circularize prior to integration. The presence or absence of TSDs across PLV clusters defined on the basis of gene-sharing patterns also corresponds to the encoded integrase.

Rve-INT was identified as the principal integrase in the *Rhodomonas*-dominated cluster C3 (85%—260 of 305) and was also common in cluster C2 (71%—68 of 97). Consistently, TSDs flank 58% of PLVs in C2 (57 of 97) and in C3 (179 of 305). In cluster C1, only two out of 298 PLVs were found to encode rve-INT. Among them, a single one was flanked by TSDs, which are 7 bp long and contain one mismatched base (TGAATAA and TCAATAA). The same type of integrase was present in approximately half of *Cryptomonas* PLVs in cluster C4 (51%—94 of 183), where 23% of members (44 of 183) included TSDs with variable sequence conservation (Supplementary Table S4). In clusters C5 and C6, which contain mixtures of *Rhinomonas* and *Storeatula* PLVs, rve-INT is rare and always fused within proteins that include at least one reverse transcriptase (RT) domain. This suggests that, in these cases, the rve-INT is encoded by nested retrotransposon insertions rather than being an inherent component of PLV genomes.

YREC-like tyrosine recombinases were present in PLVs across most analyzed cryptophytes, except for *R. baltica*. In cluster C1, YREC is the representative integrase, being present in 78% of PLV genomes (232 of 298). The same gene is also commonly found in members of C2 (15.8%—15 of 95) while being absent in cluster C3. A small fraction of YREC-encoding PLVs in cluster C1 (4.3%—10 of 232) are flanked by putative TSDs, while in C2 only two such instances were present. Closer examination of these sequences revealed that they are longer (7–23 bp) than those typically generated by rve-INT. Additionally, they often show partial sequence symmetry or have one sequence of the pair offset from the terminal inverted repeat by 1–3 bp (Supplementary Table S4). YREC is rare in cluster C4 PLVs (*n* = 6) and is restricted to peripheral members with ambiguous affiliation. TSD-like sequences were observed in clusters C5 (4.6%—5 of 109) and C6 (1.5%—1 of 66) among YREC using PLVs. However, in these cases, the sequences were generally shorter than 6 bp and likely coincidental. The unique 6-bp TSD-like sequence detected among the PLVs in these two clusters (*n* = 175) is of low complexity (GAGGG), suggesting it is a spurious match rather than a true integrase-generated TSD.

Overall, genes encoding for rve-INT domains co-occurred with YREC in 11 PLVs and one virophage across the entire dataset (*n* = 8300). In all cases, rve-INT was fused with reverse transcriptase in multidomain proteins, indicating their affiliation to inserted retrotransposons.

### PLVs employ diverse polymerases and helicases for genome replication

Several genes encoding DNA replication-associated proteins were identified in the recovered PLVs (Fig. 3; Fig. 4; Supplementary Table S6). The hallmark protein-primed DNA polymerase (pDNAP) typical of polintons appears to be rare among cryptophyte PLVs when considering only canonical matches to the DNA_pol_B_2 domain (PF03175). However, more sensitive homology searches uncovered a significantly larger number of candidates showing > 50% coverage with pDNAPs from eukaryotic adenoviruses. By aligning representative cryptophyte polymerase sequences (see “Material and methods”) with reference polinton and adenovirus pDNAPs [67], we found that most of these proteins retain conserved amino-acid motifs essential for polymerase activity (see Supplementary data in Zenodo).

Phylogenetic analysis of pDNAP proteins reveals that most sequences from *R. lacustris* (*n* = 234) and *C. borealis* (*n* = 30) cluster closely with polB1 homologs from polintons present in *Phytophthora infestans* (Oomycota), *Entamoeba invadens (Amoebozoa)*, and *T. vaginalis (Metamonada)* (Supplementary Fig. S5, Zenodo). A smaller set of pDNAPs recovered from *Storeatula* (*n* = 2), *R. nottbecki* (*n* = 3), and *Cryptomonas* (*n* = 3) groups with sequences from *G. theta* and *H. foetidus* (*n* = 3), with Group 2 Polintons (*n* = 18) forming a basal lineage to this clade. In contrast, all pDNAPs from *O. danica* (*n* = 41) form two distinct clusters that flank polymerases of *Mavirus*-type virophages.

The distinct phylogenetic placement of these pDNAPs suggests multiple gene acquisition and exchange events that do not necessarily reflect the evolutionary relationships of their protist hosts (e.g., *H. foetidus* and *Rhinomonas/Storeatula*). This hints towards extensive horizontal gene transfer across lineages, as also observed in some cases in polintons, where pPolB genes have been exchanged between unrelated phyla such as oomycetes, molluscs, and nematodes [63]. In addition, the incongruence between pDNAP and MCP phylogenies within PLVs indicates that replication and structural genes have followed separate evolutionary trajectories. While the MCP phylogeny tends to reflect the core morphogenetic lineage of PLVs, pDNAP genes show evidence of replacement, consistent with a modular genome architecture shaped by recombination and gene turnover.

Another large fraction of PLVs encode multi-domain, non-polB replicases that typically combine a primase/polymerase with a helicase domain. One such example is the fusion of a bacterial DNA polymerase I homolog (TVpol—transposon-virus polymerase) with a C-terminal superfamily 3 helicase (S3H). Reported in some of the earliest described PLVs and virophages [54, 62, 166, 167], this PolA(TVpol)-S3H fusion protein appears widespread among PLVs from *R. lacustris*, *R. nottbecki*, and *Storeatula* sp. (Supplementary Table S6). Within *R. lacustris*, TVpol-S3H is the dominant replication protein among cluster C1 PLVs. A related domain fusion, consisting of an archaeo-eukaryotic primase (AEP) and a S3H helicase, is also frequently found in cryptophyte PLVs and was previously noted in *G. theta * [166, 167].

A distinct multi-domain replicase, composed of a primase and a subfamily 2 helicase (Prim-S2H), was found exclusively in 20 *R. lacustris* PLVs belonging to cluster C2. Outside of this cluster, we identified only a single additional homolog with this domain arrangement (Prim-PolPriCT_2ResIII) in a PLV-related element (MELD virus) recovered from a metagenomic sample (GenBank: BK066479).

Instead of pPolB, some cryptophyte PLVs encode helicases assigned to superfamilies 1 or 3 (S1H and S3H, respectively) that are involved in viral genome replication [166, 167]. S1H helicases are particularly abundant in *C. borealis* (*n* = 69) and *R. lacustris* (*n* = 100) PLV genomes. In contrast, S3H helicases were only occasionally encoded independently (i.e., non-fused) across cryptophyte PLVs, with most representatives (*n* = 10) in cluster C1 of *R. lacustris*.

### Homing endonucleases

GIY-YIG and HNH homing endonuclease genes (HEGs) were identified in PLVs and related elements analyzed here, occurring both as free-standing genes and as fusions with protein domains typically involved in DNA replication. HEGs are ubiquitous selfish genetic elements that can propagate within essential host genes without disrupting their function, often by associating with self-splicing introns or inteins (i.e., peptides that self-excise post-translation) [122]. They spread by cleaving related loci lacking the HEG sequence, triggering homologous recombination that eventually generates a copy of the HEG at a new location [12, 21, 148].

Both GIY-YIG and HNH genes are well-documented in bacteriophages [8, 38] and have been observed in giant viruses [33, 102], as well as in PLVs and related elements [34, 79, 149, 166, 167]. GIY-YIG endonucleases, including members of the T5orf172 subfamily (PF10544), were widely distributed across PLVs and virophages analyzed here, mostly as standalone genes. A notable exception is the fusion between an SF1 helicase and a C-terminal GIY-YIG domain in large (~ 1000–1400 aa) proteins found in a restricted subset of Cryptomonas PLVs (*n* = 16). This domain architecture has been noted before in helicases from PLV-related Tlr1 elements found in the genome of ciliated protist *Tetrahymena thermophila* [34, 79].

HNH family HEGs were less frequent, predominantly fused with other protein domains rather than as free-standing genes, and were largely absent from YREC-recombinase-encoding PLVs. Similar to GIY-YIG HEGs, HNH units were fused to helicase domains, albeit N-terminally, in *Rhodomonas* PLVs (cluster C1, *n* = 94), a subset of *Cryptomonas* PLVs (C4, *n* = 9), *Symbiodinium* PLVs (*n* = 12), and a single PLV from the ochrophyte *H. foetidus*. In cluster C1 *R. lacustris* PLVs, this type of helicase gene is encoded immediately upstream of the rve-INT integrase, implying a functional relationship. HNH endonucleases co-occurred with YREC recombinases in only two instances. In *Symbiodinium* sp. strain CCMP2592 (contig CAJNDT_322), an HNH_3 domain was fused between NUMOD4 and AP2 domains, with sensitive searches revealing a confident match to the DNA-nicking endonuclease I-HmuI (HHpred: probability 99.89%, e-value 4e-22) [80],B. W. [135]. In *C. parva* Curly PLV, an HNH_3 endonuclease is encoded immediately upstream of the DNA packaging ATPase gene. This arrangement is similar to its positioning upstream of terminase genes in bacteriophages, where HNH endonucleases facilitate viral genome packaging within the capsid [65]. The analogous genomic context suggests a similar role for HNH in Curly PLV, likely contributing to viral genome processing before encapsidation. Alternatively, the presence of an adenine DNA-methyltransferase gene in the same genome [149] suggests that HNH may function here within a restriction-modification system, potentially mediating interviral competition or counteracting host defenses.

### PLVs use bacteriophage-like fiber proteins to infect host cells

Genes encoding for bacteriophage-like viral fiber proteins have been reported before in PLVs [63, 128, 145] and were even confirmed to be expressed under experimental conditions by the *P. globosa*-infecting PLV named Gezel-14 T [128]**.**

In particular, genes encoding the domain Peptidase_S74—chaperone of endosialidase (PF13884) are ubiquitous across PLVs recovered from most eukaryotic groups, with the exception of ochrophytes, parabasalids and amoebozoans. Single copies of the chaperone of endosialidase were often located at the C-terminus of large modular proteins (up to 3.9 k amino acids) containing repeated extracellular matrix-binding domains such as Laminin G_3 (PF13385), collagen-like repeats (PF01391), avian adenovirus fiber (PF06536), phage tail repeat-like domains (PF12789), and lower baseplate proteins (PF18338) (Supplementary Fig. S6).

These modular repeats typically form homotrimers and assemble into long fibers that attach non-covalently to the penton (mCP) component of viral capsids [112, 130]. Viral fiber proteins of animal adenoviruses are the first components to interact with cell surface integrins during the infection process [130]. Multiple cell surface-binding domains ensure increased affinity over single monomers, while their sequence determines host specificity together with more selective receptor interactions [50, 158].

Carbohydrate-binding laminin_G_3 (PF13385) domains of the concanavalin A-like lectin/glucanase superfamily were present as modular proteins in *R. nottbecki* (*n* = 23) and *Storeatula* sp (*n* = 54) PLVs and only as monomers in chrysophytes *H. foetidus* and *P. lacustris*. Within modular fiber proteins, laminin domains assembled in arrays with as many as seven repeats, frequently fused to a terminal chaperone of endosialidase domain (Peptidase_S74) or encoded in their immediate vicinity (Supplementary Fig. S6). The chaperone of endosialidase domain (Peptidase_S74) is frequently found associated with phage tail spike endosialidases and is necessary for maturation and activity of the endosialidase [132]. It cleaves itself after the maturation (frequently oligotrimerization) of the endosialidase or other proximal domains [92]. In a few cases present in *R. nottbecki* and *Storeatula*, laminin and chaperone of endosialidase units were fused or adjacent to one N-terminal domain of avian adenovirus fiber protein. Avian-type fibers determine virulence and are capable of binding in pairs to a single penton base [58]. The prototypical avian fiber proteins bind to cell surface coxsackievirus-adenovirus receptors (CARs) [151, 156] while laminin G_3 binds specific structures of exposed oligosaccharides with varying degrees of affinity [131]. Combining two types of cell binding modules (i.e., avian fibers and laminin) and harboring two fibers per penton is expected to enhance both affinity and specificity, thus increasing the fitness of their encoding PLVs.

Collagen-triple helix repeat domains were present in one to two copies fused with C-terminal chaperone of endosialidase in *R. lacustris* PLVs (*n* = 23), with two examples including N-terminal avian adenovirus-type fiber domains (Supplementary Fig. S6). In *T. vaginalis* PLVs (*n* = 20) and one organic lake virophage included in this study (HQ704801), single collagen-like domains were fused to as many as seven phage tail repeat-like domains (PTR). Among noncellular entities, collagen genes were previously reported in mimiviruses [94], virophages [81, 164], pathogenic viruses [154, 168], and in bacteriophages [4]. Although distinct from vertebrate collagen, the prokaryotic version found in viruses retains the essential molecular characteristics [48]. Prophage-encoded collagen from pathogenic bacteria indicates a protein morphology suggestive of a role as trimeric phage tail proteins involved in phage particle attachment to target bacterial cells, either directly or in combination with other tail proteins [48]. Such examples were also identified in this study. Multiple types of extracellular collagen receptors are described for eukaryotes [39], among which collagen-binding integrins (α1β1, α2β1) are known to be utilized by viruses for cell entry [60]. We assume that the collagen domains in PLV fiber proteins mainly play a role in cell attachment and infection rather than having a principal structural role. Phage tail fiber repeat domains (PTR) were either associated with collagen, as previously mentioned, or encoded separately adjacent to phage tail assembly chaperone proteins in *R. lacustris* PLVs (*n* = 19). Only one example of two PTR repeats fused to a C-terminal carbohydrate-binding H_lectin domain was found in a PLV of *Cyanophora paradoxa*. Similar phage tail-like proteins have been reported before in PLV Gezel-14 T infecting marine haptophyte *P. globosa* as well as in other eukaryotic viruses [128]. Discoidin (F5_F8_C), another non-catalytic carbohydratebinding domain [99, 143] is present mainly in *Rhizaria* (*n* = 23) and *Glaucosystophyceae* (*n* = 11) PLVs, where it occurs either individually or in pairs, or fused to patatin-like phospholipase, an enzymatic domain which has the ability to cleave fatty acids from membrane lipids [100]. One YadA head domain (Yersinia adhesin A), a known component of invasins used by eukaryote-infecting bacteria [57], was found in a single *P. micropora* PLV flanked by discoidin domains. The combined function of carbohydrate binding and fatty acid removal strongly hints towards a role in cell entry or even endosomal escape.

A remarkable fiber architecture was noted in over 30% (*n* = 411) of PLVs recovered from rhizarian *P. micropora,* where chaperone of endosialidase domains are C-terminally fused or adjacent to N-terminal lower baseplate proteins (BppL_N). These domains form trimers within a large multiprotein complex known as the baseplate in phage tail fibers, which includes the receptor binding proteins allowing specific recognition of saccharide receptors on the target cell's surface [10]. Aside from the inventory of bacteriophage-like fiber proteins reported here, deep homology searches reveal that most PLVs encode long, multi-domain proteins (> 1000 amino acids) with at least some degree of similarity to cell attachment proteins and phage fiber domains, often associated with C-terminal chaperone of endosialidase. The presence of fiber-associated homolog suggests that PLVs encoding these genes independently recognize, attach, and enter the host cell. This is consistent with previous findings regarding the isolated Gezel-14 T, which is also inferred to enter its host independently. In contrast, the Sputnik virophage relies on the surface fibers of its co-infecting giant virus for cell entry [128].

Scans for SLATT domains revealed the widespread occurrence of these putative pore-forming proteins [20] across cryptophyte PLVs (*n* = 868) (see Supplementary Table S6). While reported before in PLVs [128] and shown to cooperate with other functional domains in bacteria [20], information regarding their exact role is limited to only a few hints that link them to cell-suicide and DNA transport functions [20]. This last role implies that SLATT pores may facilitate the transport of DNA generated by the reverse transcriptase of self-replicating retroelements (i.e., retrotransposons) outside of host bacteria [20]. By analogy, we hypothesize that PLV-encoded SLATT proteins may facilitate pore-based endosomal escape of PLV DNA during host infection [105, 130]. This mechanism would involve pore formation within the membrane of late endosomes containing endocytosed PLV particles, leading to the release of viral genetic material into the cytoplasm. Pore-based endosomal escape serves as an alternative to endosome disruption by membranolytic enzymes [162] [41, 162] and was well described for some RNA viruses [1, 117, 138]. An additional clue that implies pore formation as the principal endosomal escape strategy for cryptophyte PLVs is the absence of membranolytic lipase-encoding genes, contrasted by the abundance of SLATT genes within this same group (Fig. 3; Supplementary Table S6). The ubiquitous, yet uncharacterized protein Tlr6f (PF19058—DUF5754) known to be conserved across many DNA viruses (i.e., PLVs, NCLDVs, bacteriophages) and associated with roles in viral reproduction [166, 167] was present in a large fraction of cryptophyte PLVs, often in the vicinity of SLATT domain genes (Supplementary Table S6).

DNA-modifying methylase genes were rare in cryptophyte PLVs, with the notable exception of *C. borealis* where 24 out of 177 PLVs encode for Dam-like N6 adenine-specific methylases. Their activity likely protects the PLV genome from being cut by restriction endonucleases encoded either by their competing giant viruses, known to carry restriction-modification systems [64, 155], or by the host itself as a non-specific antiviral protection strategy.

Ferredoxin-like domains (PF18406.1; DUF1281_C) were identified in proteins of 168–188 AA encoded by single-copy genes occurring across 21 *R. lacustris* PLVs from cluster C1. Ferredoxin (Fd) electron carriers were previously reported in phages of marine cyanobacteria (i.e., cyanophages) where experimental evidence indicates they show a preference for host oxidoreductases over phage-encoded ones for energy transfer [25]. It was hypothesized that Fd thus supports the metabolic reactions driving the biosynthesis of sulfur-containing amino acids, ultimately increasing the fitness of their host [25]. If true, this mechanism would also ensure the propagation of endogenous, prophage-like PLVs together with their host.

Notably, multiple PLVs recovered here are interrupted by nested GC-rich mobile elements (~ 4 to 9.5 kb) encoding reverse transcriptase and often being flanked by direct repeats (DR) of a few hundred base pairs (Supplementary Fig. S7). In different PLVs, such retrotransposon-like elements are nested either in the intergenic space or within protein-coding genes which they disrupt. Aside from GC content preferences, their presence in the small fraction of non-coding intergenic regions could be explained due to purifying selection by gene deleterious effects [55]. When disrupting the reading frame of critical genes (e.g., integrase, tail fiber proteins), retrotransposon insertion is expected to abolish the biological activity of the encoding PLV. While such insertions were noted before in PLVs and virophages [55, 166, 167], the interplay between protist host, giant viruses, PLVs/virophages and their nested retrotransposons remains convoluted.

### PLV capsids are expressed in culture and the environment

To explore whether capsid genes from endogenous PLVs are actively expressed, we queried transcriptomes from the 11 protist strains central to this study using our collection of MCP HMMs. Although PLV gene activity is not generally expected under standard culture conditions, especially for elements that depend on co-infection with giant viruses, we identified nine MCP-encoding transcripts across several cultured strains (Supplementary Table S7). High-confidence matches were found in *H. foetidus* (*n* = 2) and *R. lacustris* (*n* = 1), with additional lower-confidence matches in *C. borealis* (n = 4), *H. foetidus* (*n* = 1), and *R. lacustris* (*n* = 1). Additional genes were co-expressed with MCPs in most transcripts (Supplementary Table S7), but only a few, such as mCPs and alpha-1,2-fucosyltransferase, could be annotated. These findings complement a previous large-scale screen of protist transcriptomes from the GenBank TSA database, which identified 600 expressed MCPs across diverse eukaryotic lineages, including *Cryptomonas curvata* (*n *= 13), *C. paramecium* (*n* = 9), and several chrysophytes [13]. Notably, none of the MCPs expressed in cryptophytes, either in our study or the previous one, was of the virophage type (*Maveriviricetes*). Observed MCP expression also contrasts with the case of *T. striata* virus N1 (TsV-N1), where only a single uncharacterized PLV gene was expressed under similar culture conditions[111]. Moreover, scanning of the complete genomic assemblies (i.e., including non-eukaryotic DNA) from all 11 strains revealed no evidence of giant viruses, either as free particles or integrated in the protist host genome as giant endogenous viral elements (GEVEs).

To tentatively pinpoint the endogenous PLVs responsible for MCP gene expression in culture, we mapped the transcribed MCP sequences to PLV collections recovered from their respective protist host genomes. This analysis identified seven candidate PLVs in *R. lacustris*, two of which were exact matches (Supplementary Table S8). Notably, all matched PLVs contain well-conserved TIRs (≥ 97% identity), most exhibit 6-bp target site duplications (TSDs), and all belong to network cluster C3 (GKS1), suggesting relatively recent integration. Close matches between expressed MCPs and endogenous PLVs were also found for *H. foetidus* (*n* = 2) and *C. borealis* (*n* = 22).

High abundances of cryptophytes, particularly *Cryptomonas* and *Rhodomonas* strains, have been well documented during spring and summer phytoplankton blooms at our target study site, the Římov Reservoir in the Czech Republic [68, 141]. To determine whether PLV genes are transcribed in the environment, we queried a metatranscriptomic dataset generated from this site during one of our previous studies [19] against the newly constructed MCP HMMs. This sample represents a single time-point snapshot (16 Aug 2020) capturing combined epi- and hypolimnion transcriptional activity during the summer phytoplankton bloom. We identified a total of 60 candidate PLV MCPs, ten of which show high coverage (> 80% of both HMM model and target protein; Supplementary Table S9). One virophage-type MCP was also identified with high confidence (> 98% coverage). In several transcripts (*n *= 23), MCPs were often co-expressed with other genes, although few could be annotated using deep homology searches (see “Material and methods”). Protein domains were successfully annotated for genes co-occurring with MCPs in four transcripts: an adenovirus protease (O71070), a phage tail assembly chaperone protein (PF13368,APC_family), and SLATT_4 domains (PF18186.6) identified in two different transcripts (Supplementary Table S9). The adenoviral-type endoprotease is known to cleave Gly-Ala peptides in viral precursor peptides [152] and is essential for the maturation of several viral structural proteins [150], while the tail assembly chaperone domain, known from double-stranded DNA phages [71, 113], may facilitate viral particle assembly.

### PLV-host pair identification

To determine the protist hosts of PLVs actively transcribing MCP genes in the environmental water sample, we mapped their predicted nucleotide sequences to our collection of PLV and virophage genomes. This approach identified nine closely matching PLVs (Supplementary Table S10), all originating from the genome of *C. pyrenoidifera*. While sequence differences are expected between this cultured strain and the ones occurring at the sampling site, this method of assigning taxonomy/host is arguably more reliable in this case, considering the paucity of cryptophyte sequences in public databases, even though some sequence divergence is expected between this cultured strain and naturally occurring populations at the sampling site.

### Non-coding RNA genes in PLVs

The presence of noncoding RNAs (ncRNAs) in viruses is common, and studies of viral ncRNAs have highlighted them as regulators of their life cycle and pathogenicity [91, 153]. The relatively small genome size of PLVs (~ 20 kb) as well as viral particle sizes (~ 60 nm) imply that space is rationed, and any present ncRNA gene plays a crucial role for virus propagation. We scanned our collection of PLV/virophage genomes for the presence of ncRNA genes and identified multiple major classes: transfer RNAs (tRNAs, *n* = 558), ribosomal RNAs (rRNAs, *n* = 3), microRNAs (miRNAs, *n* = 10), self-splicing group I introns (*n* = 51), histone 3'UTR stem-loop (*n* = 6), small nuclear RNAs (snRNAs, *n* = 22), and small nucleolar RNAs (snoRNAs, *n* = 22) (Supplementary Tables S11 and S12). Within cryptophyte PLVs (*n *= 1107), these were restricted to tRNAs (*n* = 13) and group I self-splicing introns (*n* = 44). A more conservative search using Aragorn [82] revealed only ten tRNA matches in cryptophytes: one for the transfer of leucine in *C. borealis,* one for histidine in *Storeatula* sp., seven for histidine, and one for arginine in *R. lacustris*. Notably, all seven histidine-transferring tRNAs of *R. lacustris* are present in PLVs grouped within cluster C3 (GKS1) of the gene-sharing network, while the one with tRNA-Arg is unassigned. The presence of tRNAs is well known in phages [3, 103] and has previously been reported in virophages [110]. In PLVs, tRNAs likely play a role in codon compensation by facilitating the efficient translation of codons that are rare in the host genome [3]. Similar to bacteriophages, the use of alternative codons may be driven by differences in GC content between the genomes of these elements and their hosts [3, 88, 93]. One possible source of codon adaptation is the acquisition of tRNA genes from the host genome, as tRNA loci are known hotspots for viral and mobile element integration [3, 169]. However, despite scanning 1-kb upstream and downstream of all inserted cryptophyte PLVs, we did not identify any tRNA genes near integration sites.

Since viruses are known to compete for host cellular resources, PLV-encoded tRNAs may play a role in counteracting host-driven tRNA depletion [120], potentially as a response to infection by other viruses. The first sequenced giant virus infecting the same strain of *R. lacustris* (NIVA N-8/82) analyzed in this study encodes a tRNA(Arg) A34 adenosine deaminase (TadA) [155]. This enzyme catalyzes the deamination of adenosine (A) to inosine (I) at the wobble position (position 34) of tRNA-Arg, altering its codon recognition properties [165]. Given that modifying the host tRNA pool can influence codon usage preferences, it is possible that such modifications could impact the replication efficiency of co-infecting viruses. The presence of a tRNA-Arg gene in the endogenous *R. lacustris* PLV N-N882-C28_1524830-1550200 suggests a potential mechanism by which this PLV might mitigate the effects of TadA-mediated modifications, allowing it to maintain efficient translation of its own proteins. While this remains a hypothesis, future functional studies on viral tRNA modifications in *R. lacustris* could help clarify these interactions.

Group I catalytic introns (RF00028), ubiquitous ncRNA elements catalyzing their own splicing from precursor RNA [107], were found here restricted to PLVs of only a few protist groups: rhizarian *P. micropora* (*n* = 53), chlorophyte *Desmodesmus armatus* (*n* = 2), and notably, cluster C3 members of *R. lacustris* (*n* = 44). In the latter, introns (309–376 bp) are found within genes encoding a short protein (~ 50 amino acids) located just upstream of the MCP-encoding gene. While some group I introns are known to encode their own homing endonuclease that facilitates the intron’s mobility (i.e., homing) [37], this protein remains unannotated.

Different ribosomal RNA (rRNA) genes were found in three PLVs: a complete 5S gene (122nt) in dinoflagellate *P. glacialis* (PLV CAJNNW_707), a fragment of a 28S rRNA gene (203nt) in oomycete *Phytophthora quercina* (PLV JACBOW_29), and a complete 18S rRNA gene (1,334 nt) in *Phytophthora betacei* (overlapping PLVs in contig JAANHX_27). To rule out assembly errors, long reads used to assemble eukaryotic genomes harboring these PLVs were mapped and shown to span the rRNA gene insertions (Supplementary Fig. S8). In all cases, these rRNA sequences appeared to originate from the host itself. While the presence of a complete 18S rRNA in a PLV has not been observed before, this 18S rRNA gene copy was found in two adjacent PLVs that appeared to share the genes for the morphogenetic module, suggesting at least two independent insertions at this site, likely compromising the excision of both. Similarly, the presence of a fragment of 28S rRNA is also indicative of a degraded locus. In both these cases, the GC content of the PLV itself appeared also very similar to the surrounding sequence, suggesting genomic acclimatization. The 5S rRNA-containing PLV identified in *P. glacialis* is complete (18.5 kb), with intact morphogenetic genes and showing a markedly distinct GC profile compared to its host. Moreover, it is bordered by TIRs of 186 and 207 nucleotides sharing 89% sequence identity which are in turn flanked by five-nucleotide TSDs (GTTAT), characteristic of the activity of the encoded RVE integrase. The capture of fragments or even complete rRNA genes in the three PLVs is arguably a result of virus-host interactions, while it is likely the genes themselves are not functional. In general, gene acquisitions by DNA viruses, especially by those that integrate into the host genomes, are well documented [11, 75, 157].

## Conclusions

Polinton-like viruses (PLVs) were recently recognized as abundant viral particles in aquatic environments [14] and were subsequently identified as ubiquitous endogenous viruses inserted within the genomes of most sequenced protists [13]. However, the paucity of cryptophyte genomic data left this group of globally abundant algae largely unexplored, with fewer than ten PLVs recovered from *Guillardia theta* and *Goniomonas avonlea*. This work complements and expands previous knowledge regarding the diversity, genomic composition, and evolution of PLVs and virophages within representative protists with an emphasis on photosynthetic cryptophytes. To accomplish this, we leverage long-read sequencing coupled with state-of-the-art assembly methods to recover high-quality genomes for four cultured cryptophytes and one ochrophyte. Using this strategy, we reveal that over half the length of photosynthetic cryptophyte genomes consists of repetitive elements, while at the same time validating our approach by accurately assembling and spanning these long repetitive regions. For *O. danica*, we highlight the advantages of combining multiple long-read sequencing strategies, which complement extended read lengths with highly accurate reads to achieve near-complete genome recovery. We applied an ensemble of GC signature, gene marker, and terminal inverted repeat detection methods to identify more than a thousand PLVs from cryptophytes alone. This broad comparative analysis revealed that groups of vastly different PLVs can coexist and expand within the same host genome. This is particularly evident in the freshwater cryptophyte *R. lacustris*, where gene-sharing patterns and phylogenetic analyses revealed three distinct PLV populations, each characterized by unique major capsid lineages and specific genome integration and replication genes. We hypothesize that each distinct group may play a role in defending against giant viruses from similarly distant families.

Apart from conserved morphogenetic modules, gene content is highly variable even among closely related PLVs. Aside from the classical protein-primed DNA polymerase, we identified multiple examples of fusions between primase and helicase domains, which were previously recognized as derived features resulting from the replacement of the original polinton-type polymerase (Koonin Eugene V. et al., 2024). Furthermore, some PLVs harbor primases with archaeo-eukaryotic domains, similar to those present in giant viruses. We observe diverse architectures of bacteriophage-like viral fiber proteins, which likely coordinate host cell recognition and cell surface attachment. PLV-encoded fiber proteins in cryptophytes and other protists exhibit a wide array of protein domain combinations, where increased binding affinity is often associated with expanded repeats of extracellular matrix-binding domains. In some cases, fibers with avian adenovirus-like domains likely enhance interactions by binding in pairs to a single penton (i.e., mCP). Although common in ochrophytes, we did not detect any endogenized virophages (i.e., members of *Maveriviricetes*) in the comparatively large genomes of cryptophytes. While we cannot rule out the possibility that virophage sequences were missed due to limitations in genome assembly or that they encode highly divergent capsid genes not identified by current HMM models, our data do not support the presence of canonical *Maveriviricetes*-like elements in these genomes.

We show that major capsid protein (MCP) genes from cryptophyte and chrysophyte PLVs are actively expressed in culture even in the absence of detectable giant viruses, indicating that some PLVs may be autonomously active under standard conditions. This contrasts with the TsV-N1 system, where only a single uncharacterized gene was reported to be expressed [111]. Although we found no convincing evidence for the presence of giant endogenous viral elements (GEVEs), we cannot rule out interactions with undetected ones. Such GEVEs have previously been identified across a wide range of protist genomes [104] and may induce the activation of endogenous PLVs. Future studies on the co-occurrence and interplay between PLVs and GEVEs would provide more clues for the mechanisms and extent to which GEVEs influence PLV gene expression. We also report the presence of MCP transcripts in freshwater samples collected during a summer bloom event in a freshwater reservoir, with *C. pyrenoidifera* PLVs representing the principal source. These findings provide early functional evidence for potentially active PLVs and lay the groundwork for their future isolation and experimental characterization.

We identify multiple PLVs from cryptophytes and ochrophytes containing nested insertions of retrotransposon-like mobile elements that carry hallmark reverse transcriptase and are sometimes flanked by terminal repeats. Similar cases of hyperparasitism have been observed before in endogenized virophages of marine bicosoecid *Cafeteria* sp., which sometimes harbour retrotransposons of the Ngaro group (~ 6–7 kb long) [55], as well as in one *G. theta* PLV which contains a Copia retrotransposon [166, 167]. Just as virophages have the capacity to disrupt eukaryotic genes [55], nested retrotransposons may immobilize PLVs when targeting genes critical for their proliferation. In PLVs recovered here, we find examples of retrotransposon insertions both within coding regions and in intergenic spacers. In the latter case, PLVs may retain their activity and serve as shuttles for propagating nested MGEs upon their reactivation. Although unlikely, disrupted critical genes could be compensated by gene products supplied *in trans* [166, 167].

As mentioned earlier, only two PLV systems have been characterized so far, and both from marine environments [111, 128]. Experimental studies on such systems have proven invaluable in uncovering the complexities of giant virus infection mitigation by virophages [76] and more recently by PLVs [128]. The knowledge and genomic resources generated in this study, particularly regarding cryptophytes and their PLVs, combined with the recent isolation of the first giant viruses infecting *R. lacustris* [155] lay a strong foundation for establishing the first freshwater protist-GV-PLV systems, with expected broader implications for understanding freshwater cryptophyte bloom dynamics.

Despite the valuable insights provided by our genomic and transcriptomic analyses, several limitations persist that highlight the need for further investigations. Many PLV-encoded genes remain unannotated, limiting our ability to elucidate infection cycles and identify which endogenous PLVs are actively propagating. We lack definitive evidence regarding their lifestyle, particularly whether they behave like TSV-N1, capable of independent infection, or propagate more like transposons within the confines of the host nucleus. While the conserved sequences of TIRs suggest recent mobilization, it remains unclear whether PLV containing such TIRs are indeed still active. Although the cryptophyte genomes we recovered are of high quality, they are not entirely complete and remain unresolved at the level of individual chromosomes. This leaves gaps in our understanding of their full genomic architecture, which could obscure details about PLV integration patterns and local propagation.

Furthermore, beyond the limited set of expressed genes observed in our transcriptomic analyses, we lack functional evidence for most PLVs. This includes uncertainty about their lifestyle in cryptophytes—if they remain active or dormant, and whether or not they can propagate in the absence of giant viruses. These limitations underscore the need for experimental studies to determine the activity and functional roles of PLVs in cryptophyte biology. Another question that arises after observing the close semblance between endogenous PLVs of closely related *Storeatula* and *Rhinomonas* is whether they have broad or narrow host specificity.

Our genomic survey of cryptophytes provides a fundamental stepping stone for the future development of experimental studies of PLV-GV systems functioning in cryptophyte algae. Such studies will advance our understanding of PLV roles as virophage-like defensive systems acting against giant viruses and modulating cryptophyte population dynamics with broad implications in understanding the dynamics of freshwater aquatic ecosystems.

## Material and methods

### Strain cultivation and cell harvesting

Mono-eukaryotic non-axenic cultures of cryptophyte algae (*n *= 4) were obtained from the Norwegian Culture Collection of Algae (NORCCA)*: Cryptomonas pyrenoidifera* NIVA-2/81 – freshwater, *Rhodomonas lacustris* NIVA-8/82 – freshwater, *Rhinomonas nottbecki* K-1855 – brackish and *Storeatula sp.* K-1488—marine. One axenic culture of freshwater chrysophyte *Ochromonas danica* SAG 933–7 was obtained from the Culture Collection of Algae at the University of Göttingen (SAG), Germany. All cultures were grown under a light/dark cycle of 12:12 h, at 15 °C, in liquid media: *Cryptomonas pyrenoidifera* in Z8 [126] + vitamins, *Rhodomonas lacustris* in medium 20% Z8 + vitamins, *Rhinomonas nottbecki* in L1-10 [53], *Storeatula sp.* in L1 [53], and *Ochromonas danica* in Ochromonas medium (= Ochr.) [46]. Cells were harvested for DNA isolation by centrifugation: 2 L of each suspension culture in the exponential phase of growth was divided into four batches of 10 × 50 mL Falcon tubes and pelleted by centrifugation at 200—800 × g and 15 °C for 10 minutes. Washing to remove the bulk of bacteria was done 3× by resuspending cell pellets in 30 mL of freshly filtered (25 mm, Millipore, 16553 K) sterile media and using the previous centrifugation settings. Similarly, for RNA isolation, 0.5 L of cultured cells was harvested by centrifugation at 500–800 × g for 5 min at 15 °C and washed once with sterile media. After harvesting, cell pellets were resuspended in 1 ml of DNA/RNA Shield Reagent (Zymo, R1200) and stored in cryovials at −80 °C until nucleic acid extractions were performed.

### DNA extraction

Harvested cells preserved frozen (−80 °C) in DNA/RNA Shield were completely thawed at room temperature and lysed with Proteinase K (20 µl, 20 mg/mL stock; Zymo) and SDS (20 µl, 20% stock solution) for 1 h at 650 RPM and 56 °C on a thermoshaker (Grant-Bio, PHMT-PSC24 N). DNA was isolated using the Quick-DNA™ HMW MagBead Kit (Zymo, D6060) following the producer instructions for DNA/RNA Shield stored samples. The final elution of DNA from magnetic beads (2 × 33 µL) was performed twice with 120 µL of nuclease-free water. DNA concentrations were measured with a Qubit Fluorometer (version 3, Invitrogen) using the Qubit dsDNA Broad Range Assay Kit (Thermo, Q32853). Eluted DNA samples were further concentrated to meet the sequencing company’s requirements (~ 100 ng/µL) using a SpeedVac (Thermo Scientific™, DNA120) for 15–30 min at 50 °C and stored at −80 °C until dispatched for sequencing on dry ice. Separate aliquots were prepared for each sample for Illumina short-read sequencing and Nanopore PromethION long-read sequencing.

### RNA extraction

Cells stored frozen in DNA/RNA Shield were subjected to chemical lysis with Proteinase K and SDS as previously indicated for DNA extractions and with incubation time reduced to 15 min and temperature at 42 °C followed by 15 min of lysis with TRI Reagent (Zymo, R2050-1–200) added at a ratio of 3:1 v/v per sample. RNA was extracted with the Direct-zol RNA Miniprep kit (Zymo, R2050) following the manufacturer’s instructions. The final elution of silica column-bound RNA was repeated twice with 25 µL of nuclease-free water preheated at 50 °C. Eluted RNA was quantified on a BioSpec-nano spectrophotometer (Shimadzu, cat. no. 206–26300-38) followed by electrophoresis-based quality control by denaturing an aliquot of each sample (4 µL) mixed with RNA Gel Loading Dye (2×) (Thermo, R0641) at 70 °C for 10 min and followed by agarose gel electrophoresis (1% agarose in 1× TAE buffer). RNA samples passing quantity and quality requirements were stored frozen at −80 °C until shipment for sequencing.

### Long-read sequencing

Nanopore sequencing was performed in multiple batches, between 2021 and 2022, at Novogene (https://www.novogene.com/, Hong Kong, China). Libraries were prepared using the ligation sequencing gDNA kit (SQK-LSK-110; Oxford Nanopore Technologies) and sequenced on the PromethION platform (R9.4.1 flow cell chemistry). For *O. danica* SAG 933–7, additional sequencing was performed at Novogene using Pacific Biosciences (PacBio) Sequel II technology. This generated a HiFi read dataset of 17.82 Gbp with ≥ 99.8% accuracy, comprising 990,764 reads (average read length: 17,984 bp, N50: 17,853 bp).

### Short read whole-genome shotgun (WGS) sequencing

Illumina paired-end short-read sequencing (2 × 150 bp, 350-bp insert size) was performed for each strain at Novogene (Hong Kong, China) to support polishing of Nanopore long reads prior to genome assembly. Libraries were sequenced on the NovaSeq 6000 platform, with a target output of 10 Gbp per sample.

### Genome assembly

Illumina WGS raw sequencing data was subjected to quality filtration and adapter trimming by using a combination of tools from the BBMAP software pack v.38.86 https://sourceforge.net/projects/bbmap/ [22, 23]. Briefly, raw reads were interleaved and quality trimmed by reformat.sh, followed by bbduk.sh (Phred score = 18). Additionally, bbduk.sh was used to remove any remaining PhiX and p-Fosil2 contamination. Curated Illumina reads were used to generate a Burrows Wheeler Transform (BWT) dataset required for polishing noisy long-reads, according to the ropebwt2 construction approach [85]. Nanopore: Basecalling of raw sequencing data was performed at Novogene with Guppy (v. 5.0.11) using the model 2021–05-05_dna_r9.4.1_promethion_384_dd219f32. Details for basecalled FASTQ files are provided in Supplementary Table S13. Long-reads with quality > Q7 (i.e., default nanopore pass quality) were subjected to adapter and barcode trimming by Porechop v.0.2.4 [161]. Filtered long-reads were further polished using the generated Illumina BWT dataset with FMLRC2 v.0.1.8 [97] with default parameters. Polished long-reads were assembled using Flye v.2.9.1-b1780[72] (settings: –nano-corr –meta –no-alt-contigs). PacBio HiFi reads generated for axenic *O. danica* SAG 933–7 were adapter-trimmed using HiFiAdapterFilt v.2.0.0 [144]. Genome assembly was performed with hifiasm v.2.0.1[28] using curated PacBio reads together with polished Nanopore reads (*n* = 1228) filtered for a minimum length of 100 kb (longest 432 kb) as ultra-long read guides (settings: -l 3 -O 1 –primary –n-hap 4 -t 100 –ul Nanopore.100kbp.fasta). The final assembly was dereplicated using dedupe2.sh from BBMAP tools. One containment of 40 kb was detected within another contig and therefore removed. Contigs from raw assemblies were renamed according to strain ID with suffixes numbered from longest to shortest and filtered at ≥ 10 kb.

### RNA-sequencing

Illumina paired-end RNA sequencing (2 × 150 bp, 350-bp insert size) was performed following poly(A) enrichment, with a target output of 10 Gbp. All library preparation and sequencing steps were carried out by Novogene (Hong Kong, China). Raw RNA-seq reads were preprocessed using the same pipeline as described for Illumina whole-genome shotgun (WGS) data. Quality-filtered paired-end reads were assembled using MEGAHIT v1.2.9(D. [84]) (settings: –min-count 2 –k-list 29,39,49,…149 (step size of 10)). Assembled transcripts were subjected to gene prediction using Prodigal v2.6.3 [61].

### Draft genomes decontamination

Gene prediction was initially performed on draft assemblies using Prodigal v.2.6.3 [61] in metagenomic mode to detect prokaryotic DNA. Sequences derived from organellar genomes were identified by scanning predicted proteomes with hmmsearch (HMMER 3.3) [36] (e-value ≤ 1e-3) against a collection of mitochondria, plastid and nucleomorph HMMs. Mitochondrial, plastid, and nucleomorph genomes and proteins were downloaded from RefSeq. All-vs-all protein sequence comparisons were performed for sequences from each organelle separately using MMseqs2 [146]. Sequence clusters were built at 50% identity and 80% coverage, and sequences in each cluster were aligned using MAFFT [89] in L-INS-i mode, and HMM models were built with hmmbuild [36] (available in Zenodo).

Potential contaminant sequences derived from bacteria growing in non-axenic cultures were identified by scanning all predicted proteins against a custom taxonomy database consisting of GTDB release 214, UniProt (Eukaryotes and Viruses, Release 2023_01). Contigs with ≥ 50% of genes assigned to prokaryotes together with sequences derived from accessory genomes (i.e., plastids, mitochondria, nucleomorphs) were removed from draft assemblies. In the next step, assemblies were subjected to six-frame translation and taxonomy assignment with the easy-predict workflow of MetaEuk r.6-a5d39d9 [83] followed by consensus taxonomy inference by metaeuk taxtocontig (settings: majority 0.5 tax-lineage 1 lca-mode 2). Contigs assigned to bacteria with a -log(E-value) support of any taxonomic label ≥ 70% were removed as contaminants.

### Polinton MCP HMM construction

Major capsid proteins (*n* = 86) from prior publications were collected [14]. These sequences were clustered at 30% identity and 80% coverage using MMseqs2 [146]. Sequences in each cluster were aligned using MAFFT [89] L-INS-i, and HMM models were created using hmmbuild [115]. All models used in this work are available in Zenodo.

### Repeat detection and annotation

Repetitive regions were identified in draft genomes by a two-step approach involving *ab initio* prediction and homology-based searching. *Ab initio* interspersed repeat prediction was accomplished by using RepeatModeler v.2.0.3 [45] (dependencies: RECON v.1.08 [5], RepeatScout v.1.0.6 [118], rmblast v.2.11.0 + (https://www.repeatmasker.org/rmblast/) and TRF 4.09 [15]). Long terminal repeats (LTRs) were also identified with RepeatModeler by enabling LTR structural analysis using the ‘-LTRStruct’ option to run LtrHarvest [40] (dependency: GenomeTools v.1.6.2 [51]) and LTR_Retriever v.2.9.0 [109] (dependencies: MAFFT 7.487 [89], CD-HIT 4.81[90], Ninja 0.95-cluster_only[160]). The resulting library of de novo identified repeats was used together with Dfam v.3.5 (2021–10-08) (https://dfam.org/home) to perform homology-based searches throughout each genome using RepeatMasker v.4.1.2 [108].

### Non-coding RNA genes

These were identified by scanning PLV genomes (*n* = 8300) against the RFAM r.14.8 database using Infernal cmscan v.1.1.3 [106] (settings: –cut_ga –rfam –nohmmonly –tblout –fmt 2 –clanin). tRNA genes were detected by scanning with ARAGORN [82] v1.2.38 and rRNA genes by Barrnap v.0.9 [133]. To exclude the possibility of assembly artifacts, ribosomal RNA-encoding genes found within endogenous PLVs were confirmed by mapping long-read datasets used to assemble host genomes to the full-length contigs harboring them. Raw long-read genomic datasets (Nanopore or PacBio) were downloaded from EBI ENA for WGS assemblies CAJNNW01 (*Polarella glacialis*), JAANHX01 (*Phytophthora betacei*), and JACBOW01 (*Phytophthora quercina*). Raw reads were mapped against target contigs (CAJNNW_707, JAANHX_27, JACBOW_29) using Minimap2 [86] with default settings, and alignments were converted to sorted BAM files with samtools [87]. Mapped reads (> 5 kb) that spanned one or both ends of predicted rRNA genes and showed no indels longer than 50 bp were extracted and filtered using a combination of samtools, seqtk (https://github.com/lh3/seqtk), and custom bash commands (awk). Filtered reads were mapped back to contigs using the same approach, and final alignments were visualized using the Integrative Genomics Viewer (IGV) v2.16.1 [127]. Separately, the reads were converted from fastq to fasta format and scanned for the presence of non-coding RNAs against the RFAM database, as described initially for PLV genomes. Taxonomy was assigned to partial and complete PLV-encoded rRNAs using SILVA SINA online (default settings) and BLASTN against the nr database. The same rRNA genes were searched against the host genomes using MMseqs2 [146] (easy-search workflow) (Supplementary data in Zenodo).

### Genome completeness

Eukaryotic genes were predicted from assembled protist genomes using BRAKER2 [18]. Filtered paired-end Illumina RNA-seq data were first mapped to the assemblies using HISAT2 [70] in spliced alignment mode with the following settings: –sensitive –no-mixed –no-discordant. The resulting BAM files were sorted and indexed with SAMtools [87] and subsequently used as hint files for BRAKER2 to improve gene model accuracy. Genome completeness was estimated using BUSCO v5.2.2 [98], based on the presence of conserved single-copy orthologs in the predicted protein sets.

### Genetic code usage

Predicting the genetic code table (defining how mRNA codons are interpreted into amino acid sequences within sequenced organisms) was done using Codetta [139, 140]. For this purpose the five longest contigs were identified by SeqKit [137] v.0.13.2 (settings: sort –by-length –reverse) and extracted from each draft genome assembly using faSomeRecords (UCSC Executables). Results are provided in Supplementary Table S2.

### Detection of giant viruses (NCLDVs)

Complete assemblies generated in this study (i.e., including sequences from all organisms in cultures) were scanned for the presence of giant viruses using ViralRecall [2] and geNomad [24], both run with default settings.

### Data collection

Reference PLVs and virophages were collected from previously published studies: [13, 14, 111, 128, 166, 167], and (https://www.girinst.org/repbase/).

### Recovery of PLVs and virophages

Identification and recovery of PLVs and virophages inserted in genomes sequenced in this study as well as reassembled chrysophyte genomes (*n* = 5) were done based on several hallmark features: (i) the presence of polinton-like virus or virophage-type MCP genes, (ii) the presence of marker genes commonly enriched in PLVs/virophages (e.g., A32 DNA packaging ATPase, integrase, protein-primed DNA polymerase PolB, capsid maturation protease, etc.), (iii) obvious differences in GC content compared to the host genome, and (iv) the existence of flanking TIRs. First, all raw assemblies (contig length ≥ 5 kb) were subjected to gene prediction using Prodigal v.2.6.3 in metagenomic mode. MCP-encoding genes were identified by scanning proteomes with a custom-built collection of MCP HMMs (available in Zenodo) using hmmsearch (e-value < 1e-3). Marker genes commonly enriched within, but not restricted to PLVs and virophages, were identified by annotating proteomes against their specific protein domain HMMs from the PFAM v.35 database (https://pfam.xfam.org/). GC content was calculated across all contigs using bedtools nuc v2.29.2 [121] within 500 bp windows previously defined using bedtools makewindow. Output files were uploaded as GC tracks for visual inspection in a local version of the Integrative Genomics Viewer (IGV) v.2.16.1 [127]. Identification of TIRs flanking the candidate polinton-like virus regions was done using locally installed einverted [125] from the EMBOSS v.6.6.0.0 tools collection with default parameters and a maximum separation between start and end of the inverted repeat (maxrepeat) set at 80 kb. Raw results were filtered with an adhoc perl script to keep PLVs with an insert size between 6 and 80 kb and with TIR sizes of 100 bp—8 kb. Comprehensive results, including information regarding mismatches between TIR pairs, are included in Supplementary Table S4**.**

### Detection of target site duplications (TSDs)

Target site duplication (TSD) analysis was carried out on PLVs that passed terminal inverted repeat (TIR) quality filtering, using an adhoc perl script. For each PLV, 50 bp of flanking sequence on either side was extracted with the flank and getfasta functions from BEDTools [121]. Flanking pairs were then aligned using Bioperl’s Bio::AlignIO and Bio::SimpleAlign modules, which performed sliding and padding to identify consensus TSD motifs. The resulting TSD sequences are listed in Supplementary Table S4.

### PLV genome annotation and statistics

General genome statistics for all PLVs were calculated using the statswrapper.sh subprogram of BBMap (settings: format = 3 gcformat = 2). tRNA genes were predicted using ARAGORN v1.2.38 [82] with the following settings: -t -gc11 -seq -br -fasta -fo (Supplementary Table S12).

### Deep homology searches

Genes showing distant homology to CRE recombinases were identified by first constructing HHM models using known representatives from PLVs RED1 and YSL1, as well as *G. theta* elements 1 and 3. These models were generated by scanning the selected sequences against the BFD database (https://bfd.mmseqs.com/) using HHblits [123] (three iterations). The resulting HHMs (for TVpol-S3H, YREC, primase-helicase, and SLATT) were then used to search the EVE proteome with HHsearch. Search results were filtered to retain hits with ≥ 80% probability, ≥ 10% query coverage (to account for long, multidomain proteins), and ≥ 70% hit coverage to ensure recovery of full-length models. Nested mobile genetic element insertions were identified in PLV genomes based on local GC content deviations and the presence of retrotransposon-associated genes, such as reverse transcriptase (RVT) domains.

### DNA replication proteins

Candidate protein-primed DNA polymerase (pDNAPs) sequences containing PFAM domain DNA_pol_B_2 (PF03175) or showing any HHM-based homology to other virus-encoded pDNAPs (e.g., DPOL_ABEB2—UniProt, POLBc—NCBI CD) were recovered from the collection of PLVs and virophages. Other candidates were identified by manually annotating large protein clusters (≥ 30% identity, ≥ 80% coverage) using the online HHpred tool (https://toolkit.tuebingen.mpg.de/tools/hhpred) against the databases UniProt, ECOD, PFAM, NCBI_CD, and PDB. All recovered pDNAP sequences are made available in Zenodo. Polymerases from cryptophytes and ochrophytes were clustered at 80% identity and 80% coverage using MMseqs2 [146]. Protein sequences of representatives were aligned using MAFFT [89](E-INS-i mode) with related homologues recovered from the UniProt Sprot database (≥ 10% identity, min. 400 AA) and previously published pDNAP references [67]. The number of cluster members for each representative sequence is indicated next to the sequence name. The five conserved motifs defining POLB synthetic activities [17] were identified by comparison to existing annotations [67] (alignments available in Zenodo). A maximum-likelihood phylogenetic tree of pDNAPs was constructed by first aligning with MAFFT [89] (E-INS-i mode: –genafpair –maxiterate 1000) all cryptophyte (*n* = 274) and chrysophyte (*n* = 47) sequences recovered in this study with references used for a previous phylogenetic reconstruction (*n* = 68) [166, 167] and homologues of these references (*n *= 22) identified by scanning the latest UniProt Sprot (July 2024) with MMseqs2 (identity > 10%, > 400 alignment length), as well as from additional animal polintons (*n* = 8). The untrimmed alignment (*n* = 419 seqs.) was used with IQ-TREE2 v2.2.2.6 [101] to construct a phylogenetic tree using 1000 iterations of ultrafast bootstrapping and 1000 iterations of SH-alrt testing. The best fitting evolutionary model chosen using ModelFinder [66] according to Bayesian Information Criterion (BIC) was VT + F + I + G4 (Supplemenary data in Zenodo).

### PLV gene expression

Protein sequences were predicted with Prodigal [61] in assembled transcriptomes generated during this study, a metatranscriptome generated by us previously [19], as well as transcriptomes downloaded for additional representative cryptophytes and chrysophytes. Generated proteomes were scanned against our custom collection of PLV/virophage MCP HMMs with hmmsearch [115] (e-value < 1e-3) and parsed to keep only hits covering > 50% of best matching HMM models. Predicted genes (nucleotide sequences) of identified capsid proteins were retrieved and mapped with minimap2 v2.18 [86] (settings: -cx asm5) to the complete collection of PLVs and virophages. The environmental capsid genes were mapped using the same parameters. Capsid-encoding transcripts containing additional, co-expressed genes were annotated as described before.

### Network analysis

A gene-sharing (bipartite) network was constructed to assess the relatedness of cryptophyte PLVs identified in this study to each other and to previously characterized reference PLVs. Viral genomes were first dereplicated using CD-HIT [90] with a threshold of 90% sequence identity and 90% coverage. Predicted proteins from all PLVs were then pooled and clustered using MMSeqs2 [146] easy-cluster with a minimum sequence identity of 30% and a minimum coverage of 80% (settings: –min-seq-id 0.3 -c 0.8 -e 1e-3 -s 7.5 --cluster-steps 7). Protein clusters with fewer than ten sequences were excluded from the network analysis. Orthologous group (OG) numbers used in the analysis are provided in the EVE_annotations_table available in Zenodo.

A two-column table linking genomes to their shared orthologs was generated from the MMSeqs2 [146] clustering output and used to construct the bipartite network in Cytoscape v3.9.1 [134]. Node coloring was applied based on known host taxonomy. Genome and ortholog community detection were carried out using the biLouvain algorithm [114] with default parameters in sequential mode, allowing ortholog and genome clusters to be detected independently.

### Phylogenetic analysis of major capsid proteins

Reference major capsid protein (MCP) sequences were curated to include representatives of all previously defined major PLV groups and closely related viruses. The initial sequence collection was compiled from multiple sources: (1) [13, 14]; metagenomic and endogenous PLVs) [13, 14], (2) [128], (3) [111], (4) [166, 167], (5) MCP transcripts from the Marine Microbial Eukaryotic Transcriptome Sequencing Project (MMETSP) [69], and (6) animal Maverick-Polintons from RepBase (2018) (https://www.girinst.org/server/RepBase/). To organize the dataset while retaining representative sequences, MCPs from each genome in the high-quality dataset (*n* = 12,199) from [13] were clustered using MMseqs2 [146] (easy-cluster function) at 80% coverage and 90% sequence identity (settings: -c 0.8 –min-seq-id 0.9). This resulted in 3,503 sequences, which were dereplicated together using MMseqs2[146] (100% coverage and identity), yielding a simplified set of 2,949. In addition, a collection of high-quality MCPs (*n* = 1506) from the same study, which includes assignments to previously defined PLV clusters in the sequence descriptions, was added. These were further combined with MCPs from the study of [14]. Expressed MCPs detected in MMETSP were dereplicated using MMseqs2 [146] (80% coverage, 90% identity) and included. The genomes of isolated PLVs Gezel-14 T [128] and TsV-N1 [111] were retrieved, and their MCP genes were predicted and included. Virophage and Metamonada MCPs were excluded from the phylogenetic analysis due to their significant sequence divergence. For further curation, all reference MCPs were aligned using MAFFT v7.45 [89] (E-INS-i mode,–genafpair –maxiterate 1000, and phylogenetic trees were constructed with IQ-TREE2 v2.2.2.6 with default settings [101]. Phylogenetic clusters were manually pruned to simplify large clusters of closely related MCPs originating from the same species and to remove duplicate entries recovered from different sources. Additionally, MCP sequences producing extremely long branches were removed. Curation by tree construction followed by inspection and pruning was repeated several times. MCPs detected in PLVs recovered in this study using a custom set of HMM models (available on Zenodo) were filtered to retain hits with ≥ 70% coverage of both the HMM profile and the query sequence, yielding 743 PLV and PLV-like MCPs from the 1,193 endogenous virus-like elements recovered in this study. These were combined with the curated reference MCPs, resulting in a dataset of *n* = 1,483 sequences, which was aligned using MAFFT [89] (E-INS-i mode: –genafpair –maxiterate 1000). The final alignment comprised 3,882 columns with 3,597 distinct patterns, including 2,119 parsimony-informative sites, 895 singletons, and 868 constant sites. A maximum-likelihood phylogenetic tree (data available in Zenodo) was constructed from this alignment using IQ-TREE2 v2.2.2.6 [101] (settings: -B 1000 –alrt 1000 -m TEST -nstop 500 -pers 0.2). ModelFinder [66] selected Q.pfam + F + G as the best-fitting evolutionary model based on BIC. Phylogenetic clusters were labelled using previously annotated MCPs [13] included in the tree.

### Minor capsid detection

Minor capsid sequences were identified in all recovered PLVs and virophages (published and recovered here) using the HMM models published by Katzourakis et al. [7] with hmmsearch (e-value < 1e-5). Since initial results covered only a small fraction of the expected mCP diversity, we generated new HMM models in three iterative rounds. During each round, proteins from all PLVs and virophages matching the most recent mCP HMM models were extracted and clustered with MMseqs2 [146] (settings: -c 0.8 –min-seq-id 0.9). Clusters with a minimum of five mCP sequences were aligned with MAFFT [89] (L-INS-i mode), and alignments were converted to Stockholm format with sreformat (biosquid_1.9 g) and used with hmmbuild [36] to generate updated HMMs targeting mCPs.

## Supplementary Information

Supplementary Material 1.

Supplementary Material 2.

## Data Availability

The sequencing data generated in this study are publicly available in the European Nucleotide Archive (ENA) at EMBL-EBI under the project accession number PRJEB79909, including raw genomic and transcriptomic data, as well as the genomes of *Cryptomonas pyrenoidifera* NIVA-2/81 (GCA_965119555.1), *Ochromonas danica* SAG 933-7 (GCA_965119565.1), *Rhinomonas nottbecki* K-1855 (GCA_965120255.1), *Rhodomonas lacustris* NIVA-8/82 (GCA_965120265.1), and *Storeatula* sp. K-1488 (GCA_965120275.1). The assembled genome of *Cryptomonas borealis* NIES-276 (GCA_965233945.1) has been deposited in ENA under its original project (PRJDB12131, BioSample ID SAMD00399137). The genome of *Rhodomonas baltica* CCAP 979/9 (GCA_965233955.1) is also available in ENA, associated with its original BioProject (PRJEB55799, BioSample ID SAMEA8100079). Transcriptomic sequencing data derived from environmental samples are available at ENA under BioProject PRJEB35770 (run accession ERR5100021). All assembled protist genomes and transcriptomes, phylogenetic trees (MCP and PolB) and their alignments, all PLV and virophage genomes, gene annotations, HMM models and the bipartite network files are available in Zenodo (10.5281/zenodo.14540677).

## Abbreviations

PLV: Polinton-like virus
TIR: Terminal inverted repeat
TSD: Target site duplication
EVE: Endogenous viral elements
NCLDV: Nucleocytoplasmic large DNA virus
