# Supplementary material for: Lineage-specific expansions of polinton-like viruses in photosynthetic cryptophytes
Source: Microbiome. 2025 Jul 1;13:154. doi: 10.1186/s40168-025-02148-0 (PMC12220804; doi:10.1186/s40168-025-02148-0)
Supplement: Supplementary file 3 — Supplementary Material 2. [file 40168_2025_2148_MOESM2_ESM.pdf]

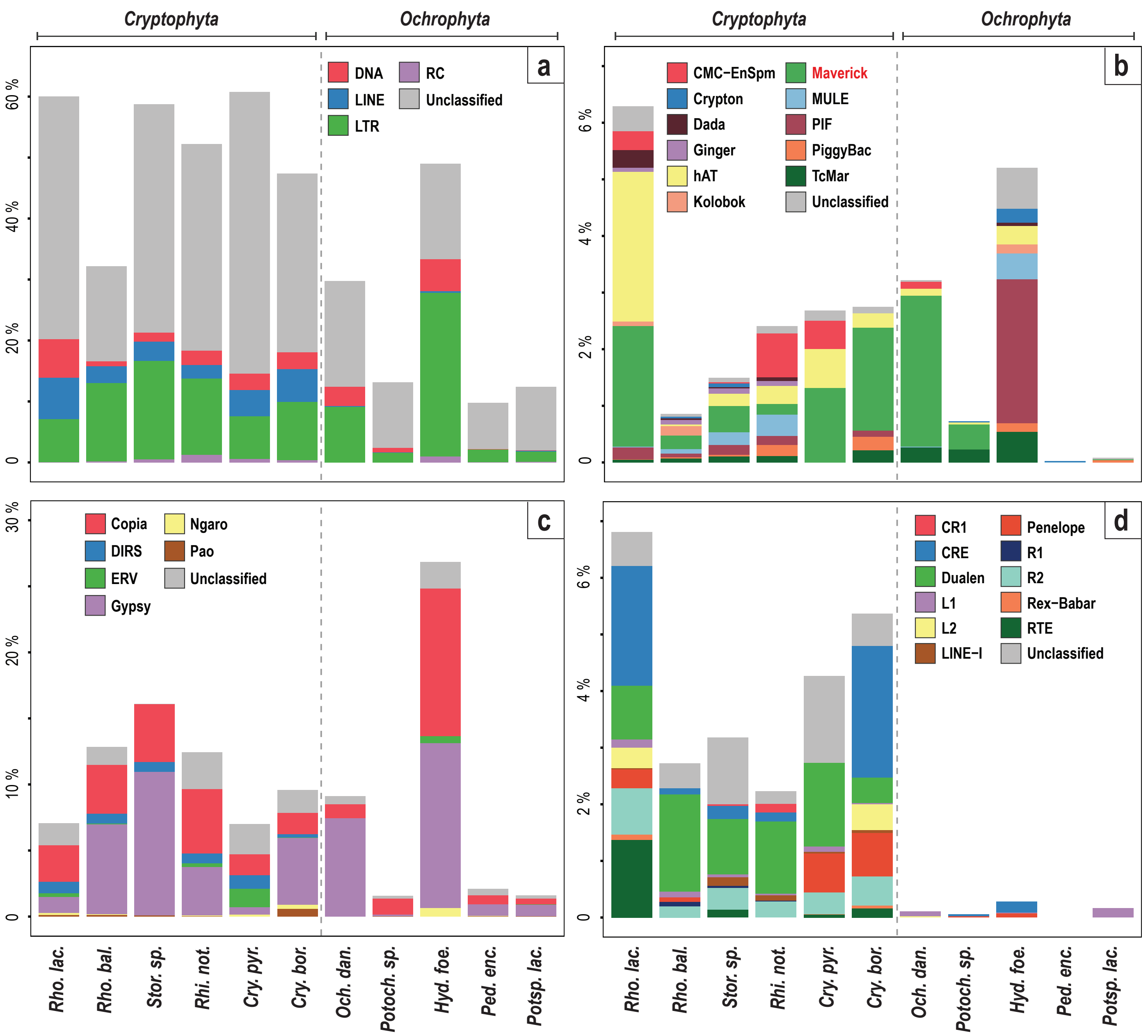

Supplementary Figure S1. Genomic repeat content of assembled *Cryptophytes* and *Ochrophytes*. **a)** Broad categories of mobile genetic elements identified by RepeatMasker within reconstructed genomes as percentage of total recovered genome length. **b)** Repeats contributed by the class of DNA mobile elements. **c)** Repeats assigned to the class of LTR (Long Terminal Repeat) elements. **d)** Repeats assigned to the group of LINE retrotransposons. In total n = 11 genomes were scanned using Repeat-Masker against the DFAM 2.2 database. Taxonomy abbreviations: *Rho. lac - Rhodomonas lacustris, Rho. bal. - Rhodomonas baltica, Stor.* sp. - *Storeatula* sp., *Rhi. not. - Rhinomonas nottbecki, Cry. pyr. - Cryptomonas pyrenoidifera, Cry. bor. - Cryptomonas borealis, Potoch.* sp. - *Poterioochromonas* sp., *Hyd. foe. - Hydrurus foetidus, Och. dan. - Ochromonas danica, Ped. enc. - Pedospumella encystans, Potsp. lac. - Poteriospumella lacustris*.

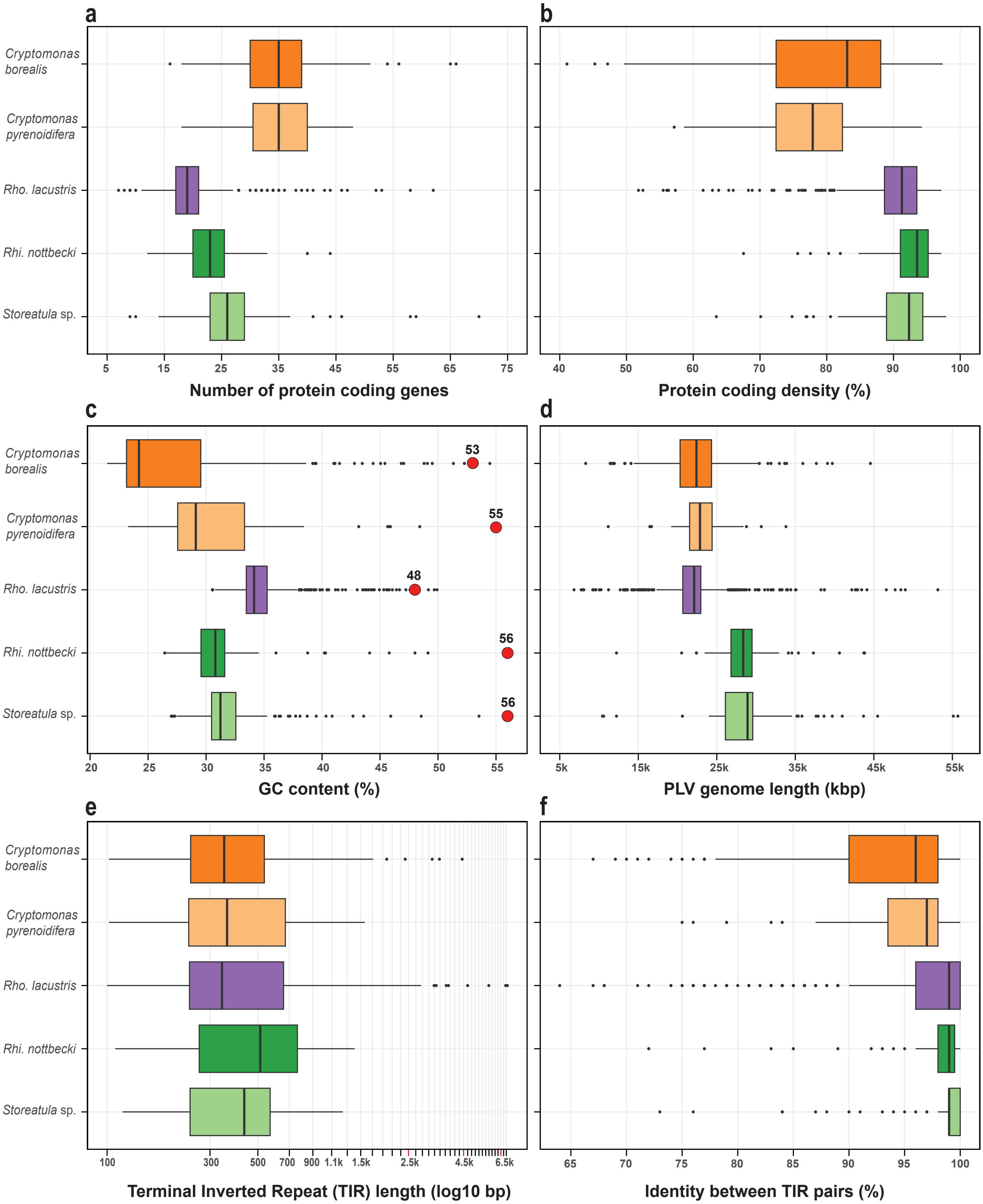

Supplementary Figure S2. Genomic characteristics of PLVs recovered from cryptophytes. Panels **(a–f)** show various genome statistics for host species with more than 10 detected PLVs. In panel **(c)**, the GC content of host genomes is indicated by red circles, representing the average GC% across the entire genome.

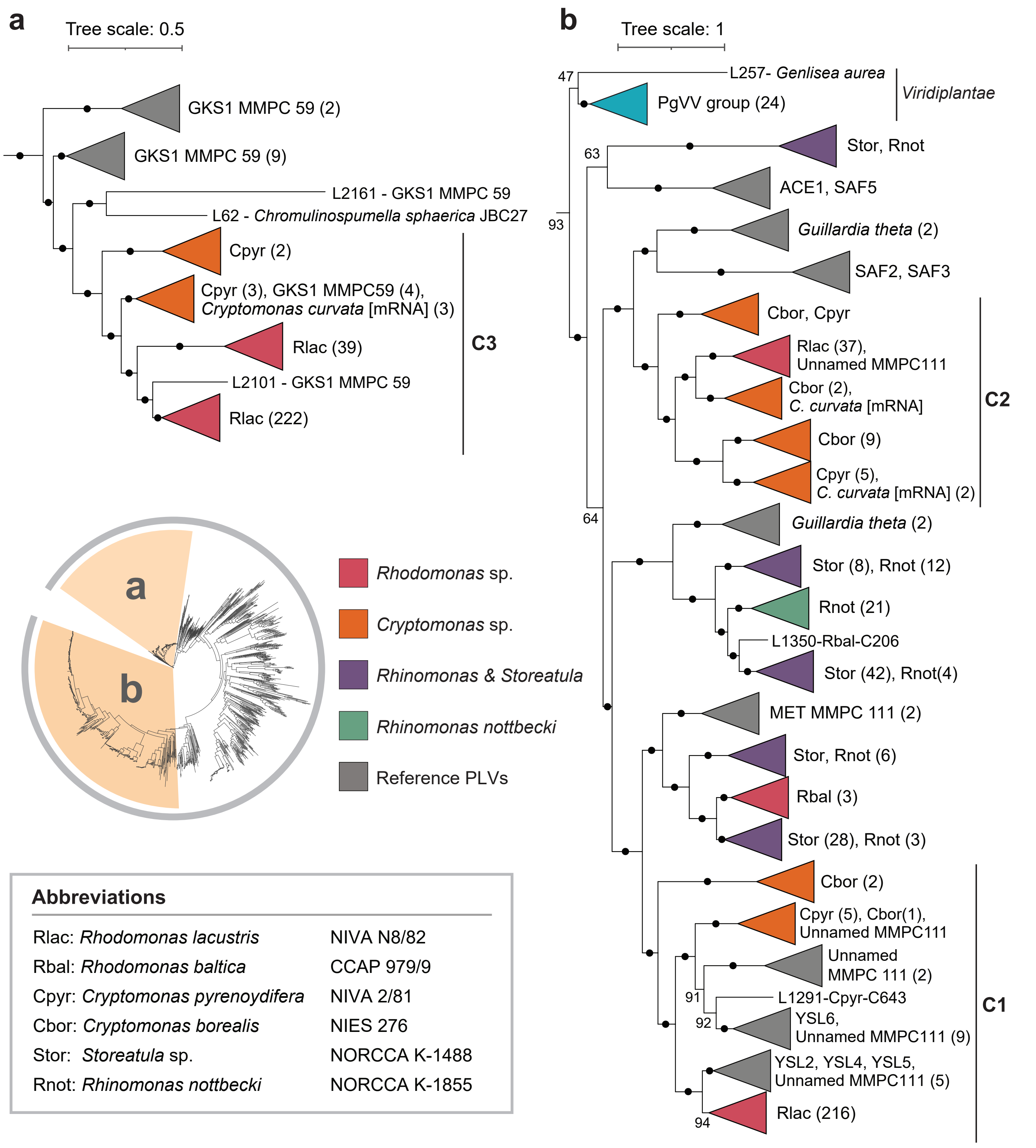

Supplementary Figure S3. Subtrees from the phylogenetic tree shown in Figure 1, depicting clusters that include major capsid proteins (MCPs) from cryptophyte-associated PLVs.
a) GKS1-type PLVs form a basal clade in the phylogenetic tree, clustering with metagenomic sequences and previously described chrysophyte PLVs assigned to this group. A significant fraction of *Rhodomonas lacustris* PLV MCPs fall within this cluster, as confirmed by matches to the specific HMM profile (M13 model). A circular diagram indicates the position of the two subtrees within the complete MCP tree.
b) A large clade of *R. lacustris* MCPs (n = 216) forms a distinct terminal cluster, adjacent to previously reported PLVs YSL2, YSL4, and YSL5. MCPs from *Cryptomonas* species are found at the base of this clade. MCPs from *Rhinomonas nottbecki*, *Storeatula* sp., and *Rhodomonas baltica* are positioned between the *Rhodomonas* and *Cryptomonas* clusters C1 and C2. Ultrafast bootstrap support is shown as black dots for nodes with UFB ≥ 95; values are indicated for branches with lower support. Clusters C1–C3, referenced in Figure 1, are also annotated here in both subtrees.

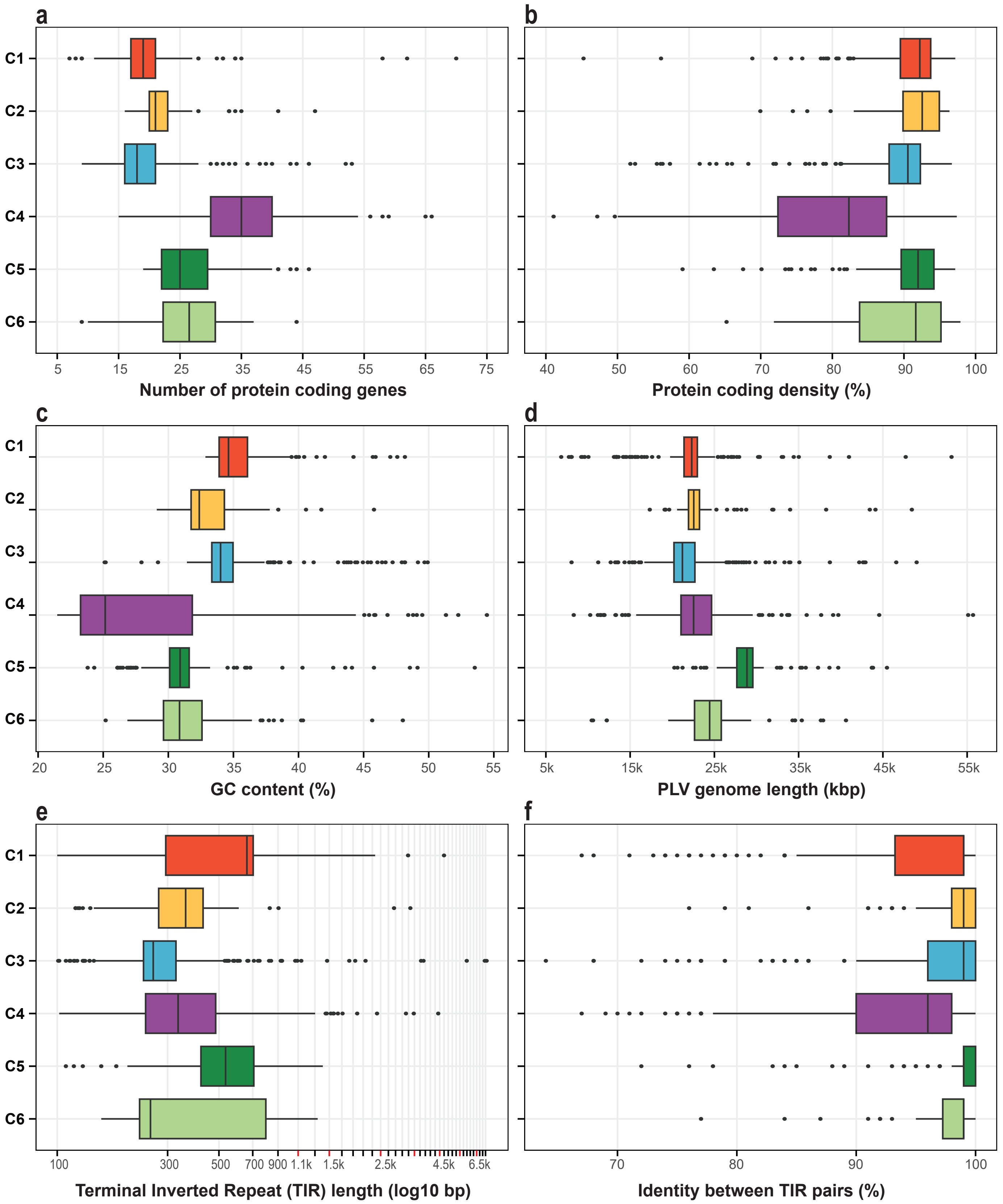

Supplementary Figure S4. Genomic characteristics of PLV clusters identified through bipartite network analysis. Colors correspond to those used in **Figure 1b** to indicate cluster membership. Outliers are shown as individual dots

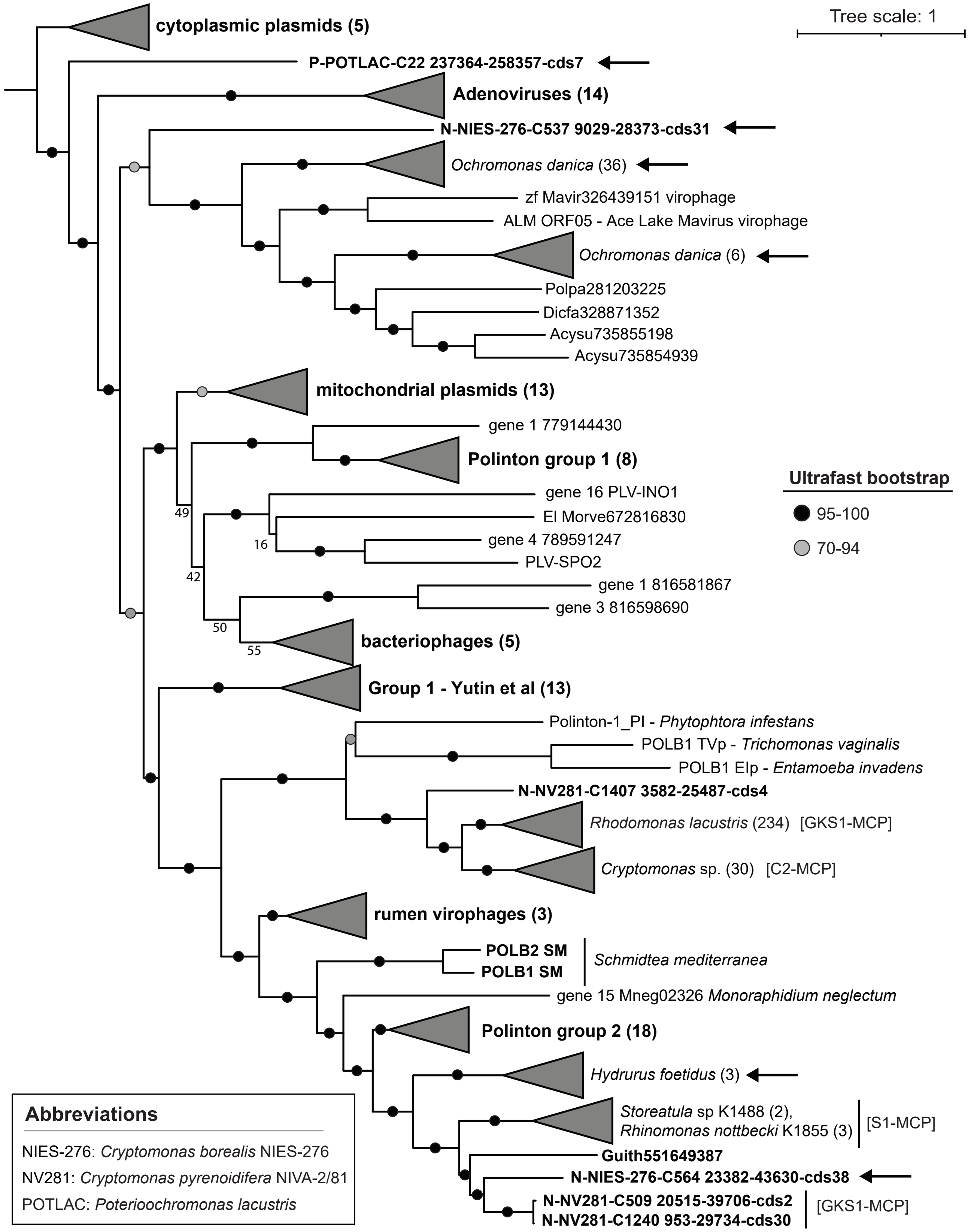

Supplementary Figure S5. Maximum-likelihood phylogenetic tree of protein-primed DNA polymerases (pDNAPs). Sequences recovered in this study from cryptophyte and ochrophyte Polinton-like viruses (PLVs) were aligned with previously published sequences from PLVs, virophages, and related elements. Corresponding major capsid protein (MCP) cluster assignments are indicated in square brackets, where applicable. Horizontal arrows denote the positions of PLV pDNAPs identified in this study that lack cluster designations. The tree is rooted with polymerases from cytoplasmic plasmids. Taxonomic abbreviations: Acysu – *Acytostelium subglobosum*; Guith – *Guillardia theta*; Dicfa – *Dictyostelium fasciculatum*; Polpa – *Polysphondylium pallidum*.

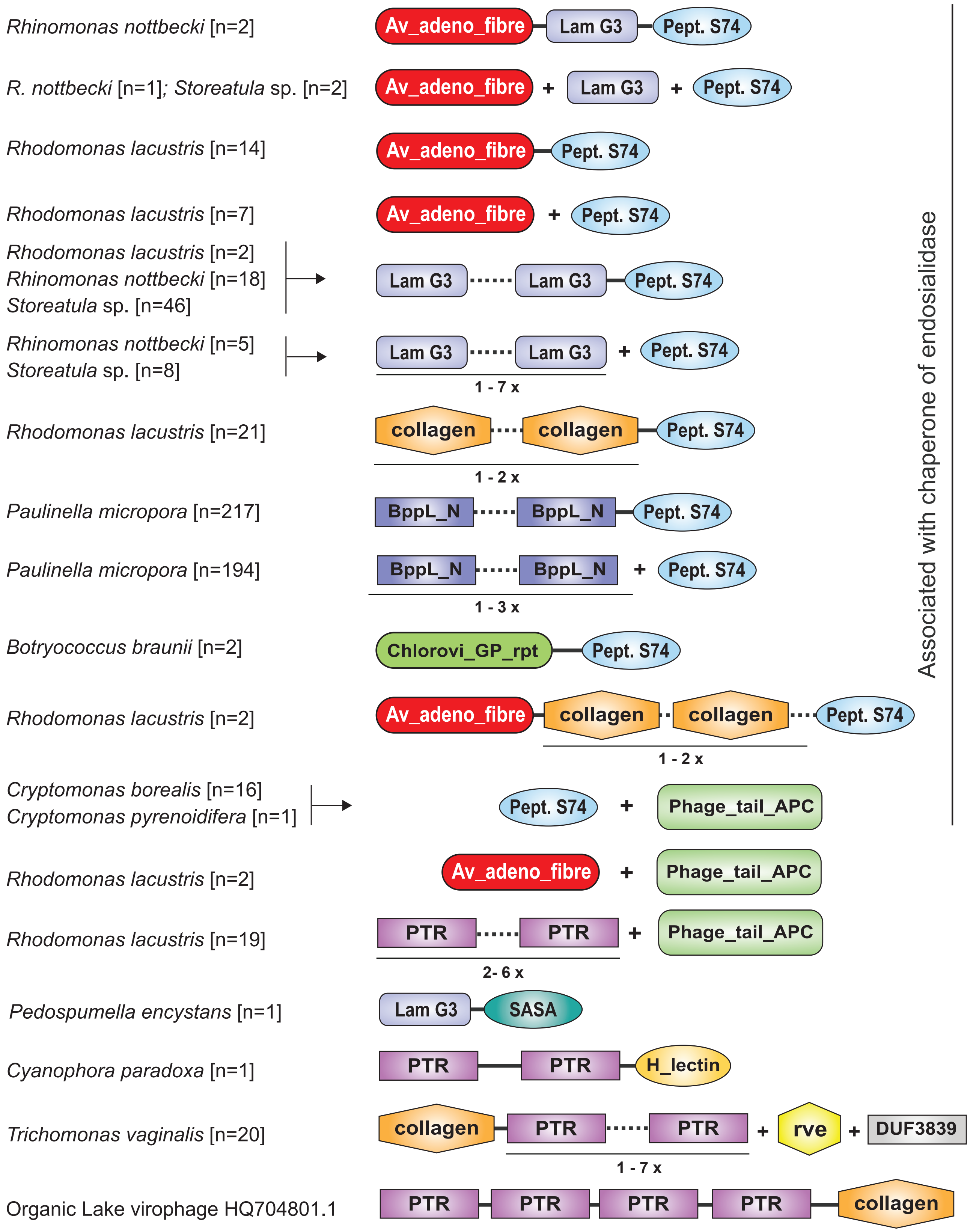

Supplementary Figure S6. Domain organization of proteins involved in cell recognition and attachment. Protein domains encoded in adjacent genes are indicated by a plus sign. Domain abbreviations: Pept. S74 (PF13884) - Chaperone of endosialidase; Av_adeno_fibre (PF06536) - Avian adenovirus fibre, N-terminal; Lam G3 = Laminin_G_3 (PF13385) - Concanavalin A-like lectin/glucanases superfamily; Collagen (PF01391) - Collagen triple helix repeat (20 copies); SASA (PF03629) - Carbohydrate esterase, sialic acid-specific acetylesterase; BppL_N (PF18338) - Lower baseplate protein N-terminal domain; Chlorovi_GP_rpt (PF06598) - Chlorovirus glycoprotein repeat; Phage_tail_APC (PF16778) - Phage tail assembly chaperone protein; PTR (PF12789) - Phage tail repeat like; rve (PF00665) - Integrase core domain.

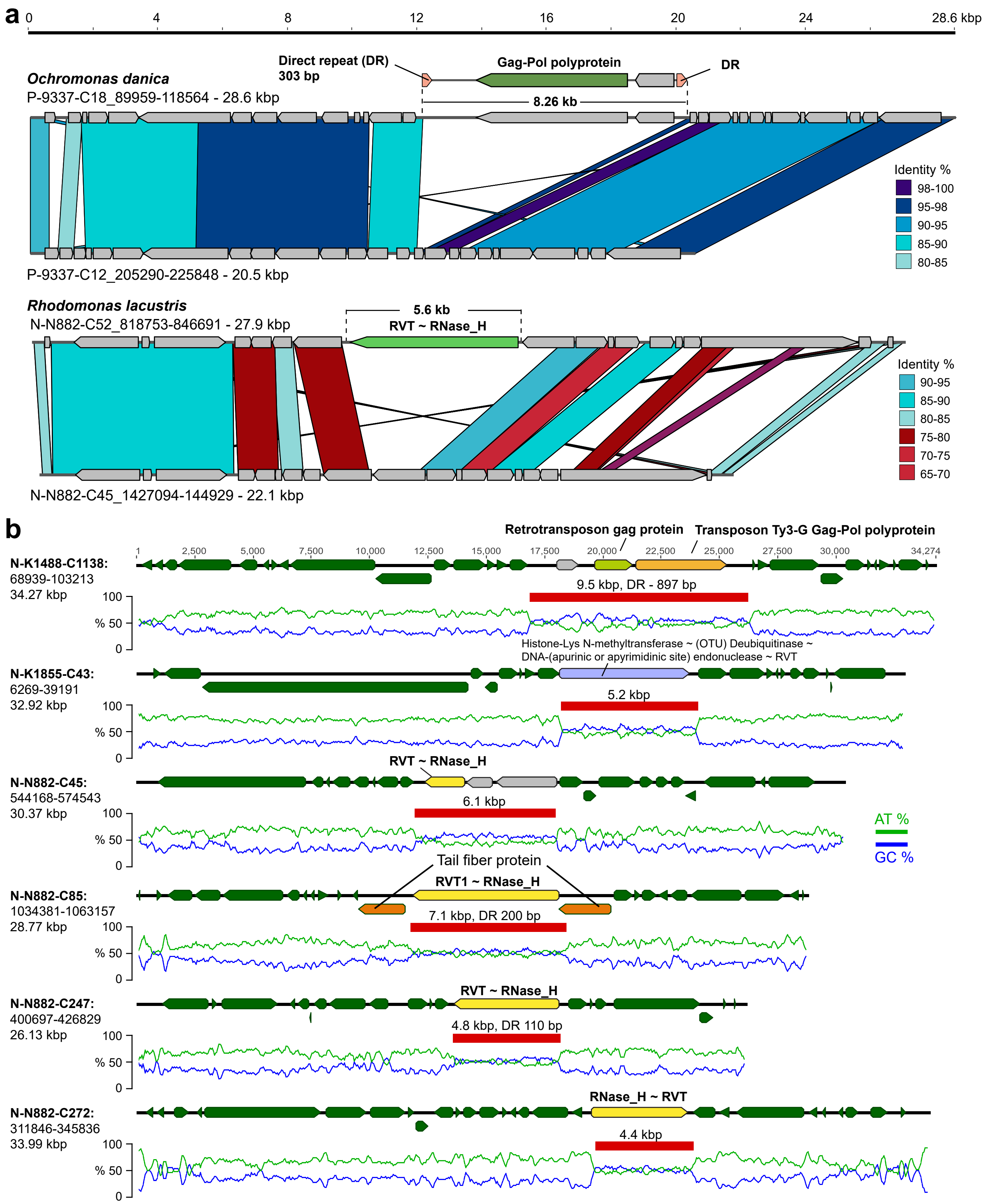

Supplementary Figure S7. Nested mobile genetic elements detected in PLV genomes.
a) DNA homology comparison between pre- and post-insertion forms of related PLVs co-exisiting in the same host genomes (i.e. *Ochromonas danica* and *Rhodomonas lacustris*). **b)** Examples of retrotransposon-like mobile elements found inserted in cryptophyte PLVs. All represented inserts encode protein domains with reverse transcriptase (RT) activity. Some retrotransposons are flanked by direct repeats (DR) while for others no flanking direct or inverted repeats could be detected. GC and AT content (%) was plotted over windows of 100 bp.

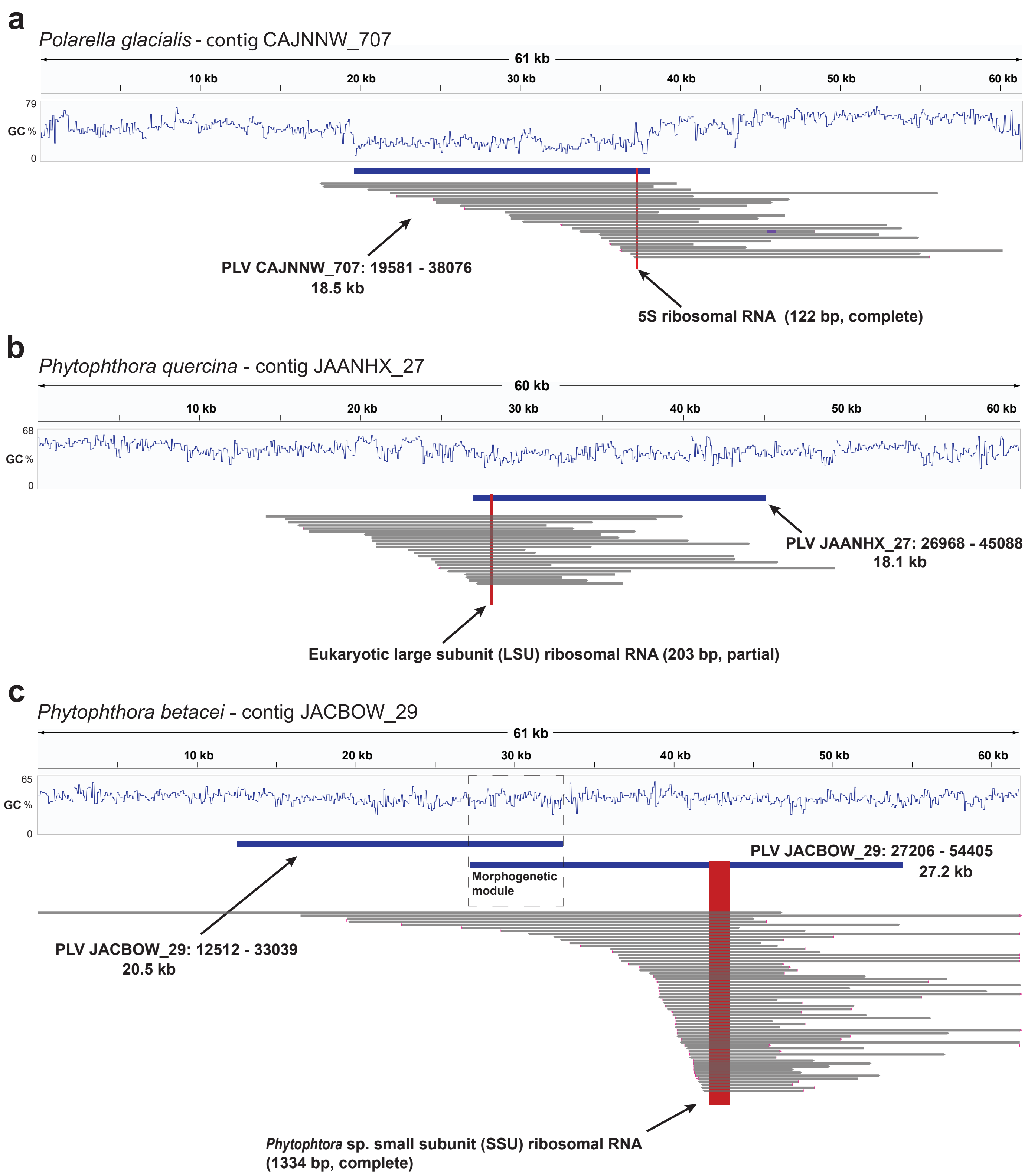

Supplementary Figure S8. Ribosomal RNA genes encoded within Polinton-like viruses (PLVs).
a) A PLV from the dinoflagellate *Polarella glacialis* (CAJNNW_707) encodes a 5S rRNA subunit.
b) In the oomycete *Phytophthora quercina*, one PLV (JAANHX_27) includes a short fragment (203 bp) of a 28S large subunit (LSU) eukaryotic rRNA gene. **c)** In the oomycete *Phytophthora betacei* (JACBOW_29), two putative PLVs overlap, sharing the defining morphogenetic module. A complete 18S small subunit (SSU) rRNA gene is included in the second candidate PLV. GC content (%) was calculated in 100 bp windows. Long reads that span complete rRNA genes were mapped back to the contigs and are shown as dark gray lines. Red columns indicate the positions of complete or partial rRNA genes.
